# Extracellular Vesicles: A New Source of Biomarkers in Pediatric Solid Tumors? A Systematic Review

**DOI:** 10.3389/fonc.2022.887210

**Published:** 2022-05-24

**Authors:** Nathalie S. M. Lak, Elvera J. van der Kooi, Agustin Enciso-Martinez, Estefanía Lozano-Andrés, Cees Otto, Marca H. M. Wauben, Godelieve A. M. Tytgat

**Affiliations:** ^1^ Research Department, Princess Máxima Center for Pediatric Oncology, Utrecht, Netherlands; ^2^ Department of Experimental Immunohematology, Sanquin Research, Amsterdam, Netherlands; ^3^ Medical Cell Biophysics Group, University of Twente, Enschede, Netherlands; ^4^ Department of Biomolecular Health Sciences, Faculty of Veterinary Medicine, Utrecht University, Utrecht, Netherlands

**Keywords:** pediatric oncology, solid tumors, extracellular vesicles, neuroblastoma, rhabdomyosarcoma, osteosarcoma, hepatoblastoma, desmoplastic small round cell tumor

## Abstract

Virtually every cell in the body releases extracellular vesicles (EVs), the contents of which can provide a “fingerprint” of their cellular origin. EVs are present in all bodily fluids and can be obtained using minimally invasive techniques. Thus, EVs can provide a promising source of diagnostic, prognostic, and predictive biomarkers, particularly in the context of cancer. Despite advances using EVs as biomarkers in adult cancers, little is known regarding their use in pediatric cancers. In this review, we provide an overview of published clinical and *in vitro* studies in order to assess the potential of using EV-derived biomarkers in pediatric solid tumors. We performed a systematic literature search, which yielded studies regarding desmoplastic small round cell tumor, hepatoblastoma, neuroblastoma, osteosarcoma, and rhabdomyosarcoma. We then determined the extent to which the *in vivo* findings are supported by *in vitro* data, and vice versa. We also critically evaluated the clinical studies using the GRADE (Grading of Recommendations Assessment, Development, and Evaluation) system, and we evaluated the purification and characterization of EVs in both the *in vivo* and *in vitro* studies in accordance with MISEV guidelines, yielding EV-TRACK and PedEV scores. We found that several studies identified similar miRNAs in overlapping and distinct tumor entities, indicating the potential for EV-derived biomarkers. However, most studies regarding EV-based biomarkers in pediatric solid tumors lack a standardized system of reporting their EV purification and characterization methods, as well as validation in an independent cohort, which are needed in order to bring EV-based biomarkers to the clinic.

## Introduction

Extracellular vesicles (EVs) are released by virtually every cell in the body (1). EVs therefore play a key role in intercellular communication and are involved in several aspects of cancer (2, 3), making cancer-associated EVs a promising source of biomarkers (4, 5).

EVs are highly heterogenous, and many subtypes of EVs have been defined based on their size, cell type of origin, biogenesis route, and the cellular processes in which they are involved (1). Intraluminal vesicles (ILVs) are formed within the endosomal network and are released by the fusion of multivesicular bodies (MVBs) with the plasma membrane; the resulting EVs are thereafter called exosomes (1). In contrast, microvesicles (MVs) are formed and released *via* direct budding of the plasma membrane (1). Other EV subtypes include apoptotic bodies, ectosomes, oncosomes, and microparticles (1, 6). Because the various EV subtypes overlap with respect to their size and composition, their classification and nomenclature remain open for debate (1, 2, 7). For the purposes of this review, however, we will use the rather general term “EVs”.

**Figure 1:**
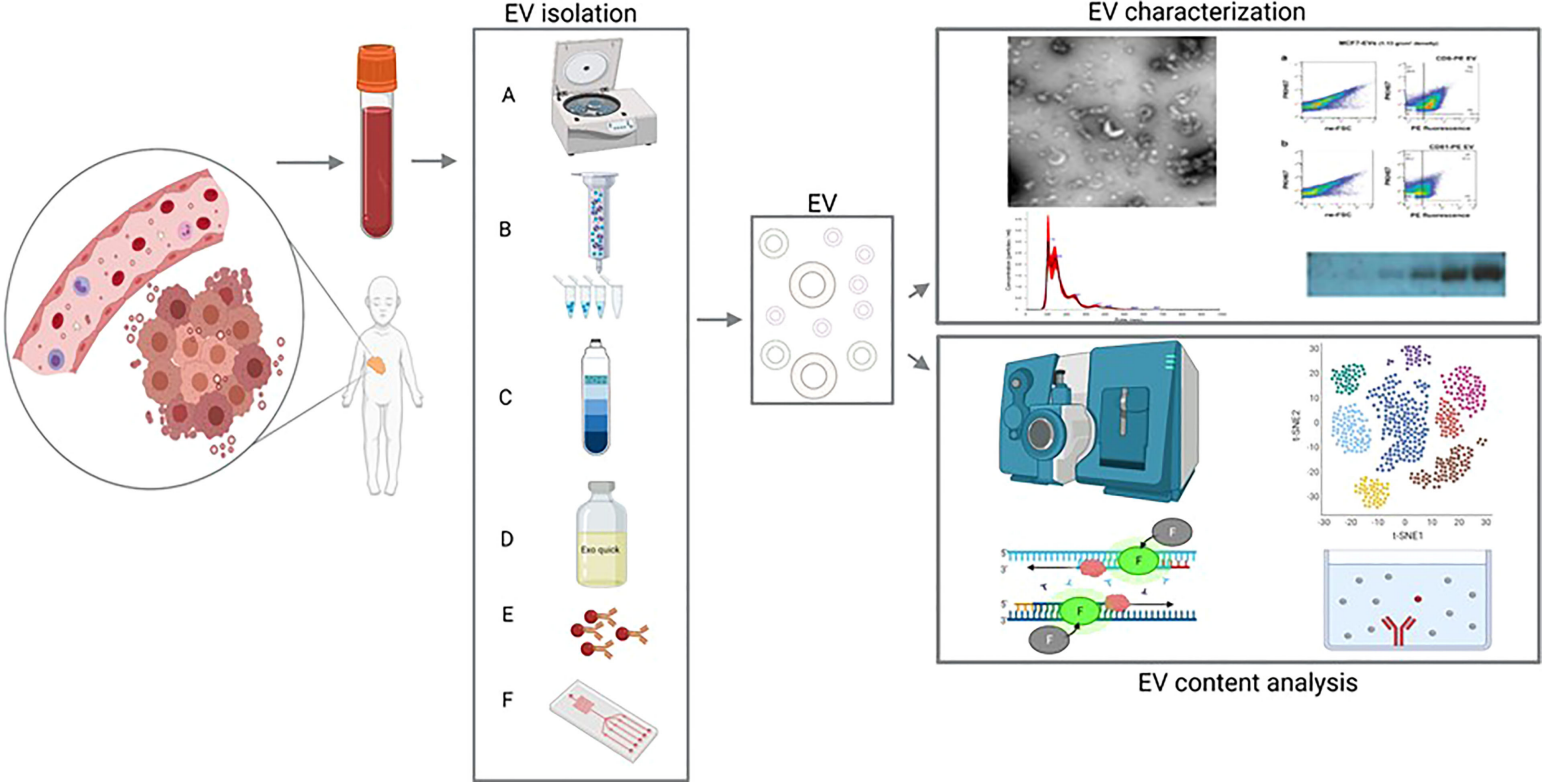
Extracellular vesicles (EVs) from blood as a liquid biopsy: isolation methods and downstream analyses. Left: EVs (including tumor-derived EVs) are isolated from peripheral blood and purified using differential centrifugation/ultracentrifugation **(A)**, size exclusion chromatography [SEC; **(B)**], density gradient **(C)**, commercially available precipitating agents [e.g., Exoquick; **(D)**], immunoprecipitation/capture **(E)** or microfluidic/nanostructure approaches **(F)**. Right, top panel: the isolated EVs are then characterized using (from the top-left, moving clockwise) electron microscopy, flow cytometry, western blot analysis, and/or nanoparticle tracking analysis (NTA). Right, bottom panel: the EV contents are analyzed using (from the top left, moving clockwise) mass spectrometry, RNA sequencing, enzyme-linked immunosorbent assay (ELISA), and/or RT-qPCR.

EVs play an essential role in both physiological and pathological processes by mediating cell-cell communication (8). The precise effect exerted by a given EV is determined primarily by its surface molecules and its cargo, which can include proteins, lipids, nucleic acids such as DNA and RNA, and metabolites derived from the cell of origin (9). Lipid encapsulation protects the cargo from degradation and allows the EV to be transported throughout the body and across physiological barriers (10). Thus, EVs can be recovered from various bodily fluids, including blood (**Figure 1**) (4, 11, 12), cerebrospinal fluid (13), urine (14), and breast milk (15). Moreover, EVs can also be isolated from liquid biopsies, providing a minimally invasive, clinically relevant method for monitoring patients with cancer (16).

In cancer, EVs play a role in both disease progression and metastasis by mediating the crosstalk between tumor cells and their environment (3, 17, 18). EVs can also induce a tumor-promoting phenotype in recipient cells (19), and EVs have been associated with the induction of multi-drug resistance in several cancer types (20). Compared to non-malignant cells, cancer cells release relatively high amounts of EVs (2, 21, 22), thus translating to higher numbers of EVs present in the blood of cancer patients compared to healthy controls. Moreover, the cargo contained in tumor-derived EVs differs from the cargo in EVs released by healthy cells, and the contents of tumor-derived EVs can change during tumor progression, reflecting the stage of the tumor (23).

Compared to other biomarkers from liquid biopsies for the use in pediatric solid tumors, EVs have some potential advantages (24). The use of cell-free DNA from plasma has been extensively studied for different tumor entities using various molecular techniques. The presence of the methylated tumor suppressor gene RASSF1A can be detected in plasma for several types of pediatric solid tumors, and can be used to monitor therapy response (25, 26). For neuroblastoma, tumor-specific aberrations in the MYCN and ALK genes (mutations and copy number alterations) can be monitored during the course of the disease (27, 28). Copy number profiling can be performed on cell-free DNA to detect a tumor-derived signal, and this can be combined with the copy number profile from the primary tumor, offering a more comprehensive overview of the genetic landscape of the tumor and its metastatic lesions (29). However, since plasma mostly contains non-tumor cell-free DNA, the signal-to-noise reduction can be challenging, especially considering that not all tumors shed large amounts of cell-free DNA (25, 30) Another option that has been explored, is detection of circulating tumor cells in blood, or bone marrow, using tumor-specific targets. This has been shown to be of clinical value in neuroblastoma and rhabdomyosarcoma (31–33). Still, it is hard to identify targets for specific tumors, especially for the detection of relapse since tumor cells can change their molecular characteristics under influence of therapy, and not all tumors shed large numbers of tumor cells into circulation (34–37). Biomarkers that are isolated from purified EVs benefit from a decrease of background noise and, since all cells in the body shed EVs, are not depending on the presence of circulating tumor cells. Furthermore, the lipid bilayer of EVs offers protection from RNAse naturally present in plasma (38, 39).

Importantly, the outcome of an EV study can be affected by the methods used to enrich (including isolation and purification) and analyze the EVs. Over the past decade alone, a wide range of methods have been used to isolate EVs, including ultracentrifugation, size-exclusion chromatography, density gradient centrifugation, precipitation, and immunocapture (**Figure 1**) (40). Apart from these conventional approaches to EV purification, microfluidic and nanostructure-based techniques have emerged in recent years. Potentially, these approaches pair high-throughput testing to low sample input, which makes them very interesting for clinical, point-of-care use. Most of these techniques depend on differences in size and/or (immuno-)labelling of the EVs (41–43).The reproducibility and reliability of EV-derived data depend heavily on the enrichment method used, as demonstrated back in 2014 by Van Deun et al. (44), who used several methods to isolate EVs from conditioned medium from a breast cancer cell line and found clear differences with respect to the number of co-isolates, EV morphology, EV quantity, and EV content. The authors found that the OptiPrep density gradient method outperformed both ultracentrifugation and commercially available precipitating agents with respect to the purity of the resulting EVs; they also found that their downstream analysis of protein and RNA content was greatly affected by the enrichment method used, thus potentially compromising the reproducibility and validation of EV studies (44). Apart from the purity of EVs, an important aspect to consider is the workflow and costs from every technique. Size exclusion chromatography and precipitation approaches are relatively rapid considering the workflow, whereas differential centrifugation requires specific material and is time-consuming, as is density gradient centrifugation. Immunocapture demands knowledge on markers present on the surface of EVs, which restricts unbiased studying of a heterogeneous EV population (40, 42).. The combination of different techniques, like size exclusion chromatography followed by density gradient centrifugation is considered as an approach for pure EV recovery. However, this is very time consuming and also results in a loss of total EV (40, 45). Various techniques for EV characterization and validation are used. Western blot is available in most laboratories and several established EV-related markers are often used, e.g. CD9, CD63, CD81 or TSG101 (40). However, this approach depends on the assumption that all EV of interest contain these markers, which can turn into a self-fulfilling prophecy. Nanoparticle tracking analysis can determine size and concentration of particles in a solution, however it does not only measure EVs but also other particles like lipoproteins or protein aggregates (40) Flowcytometry is often performed to confirm the presence of EV. This approach is prone to erroneous measurements, since detection of EVs depends on specific instrument requirements and correct interpretation of data, which can be ambiguous (46, 47).

In an attempt to improve both precision and standardization in the EV field, the International Society for Extracellular Vesicles (ISEV) published a position paper in 2014 with guidelines regarding the minimal experimental requirements for studies involving EVs (48); this was followed in 2018 by a research community−based update entitled Minimal Information for Studies of Extracellular Vesicles (MISEV) (49). Together, these guidelines provide researchers with criteria for isolating, enriching, and analyzing EVs, as well as guidelines for the standardized reporting of their findings, thus improving both reproducibility and validity, and paving the way towards the clinical application of EVs as a biomarker (48, 49). Moreover, the online crowdsourced knowledge base EV-TRACK (transparent reporting and centralizing knowledge in extracellular vesicle research; https://evtrack.org/)—to which essential information regarding methods for enriching and characterizing EVs can be published and submitted manuscripts can be uploaded—also contributes to increasing the accuracy, rigor, and reproducibility of EV research (50, 51). When a new study is submitted to EV-TRACK, a so-called EV-METRIC score is calculated and controlled by the EV-TRACK administrators for inclusion in the database, allowing other researchers to objectively evaluate the technical reproducibility and detailed reporting of the study (50, 51).

In several adult cancers, EV-based biomarkers have been shown to be correlated with both disease stage and outcome (21, 22, 52–56). Due to significant differences in pathophysiology between adult and pediatric cancers, however, this knowledge cannot simply be extrapolated from adults to pediatric patients. For example, in adults cancer progression is often driven by multiple genetic aberrations, whereas pediatric tumors have a distinct genomic landscape typically characterized by a paucity of recurrent mutations and structural variants (57–59). Furthermore, the genes that are mutated in childhood tumors often differ from those in adult tumors and tend to be specific to certain cancer types and individual patients (60, 61).

**Figure 2:**
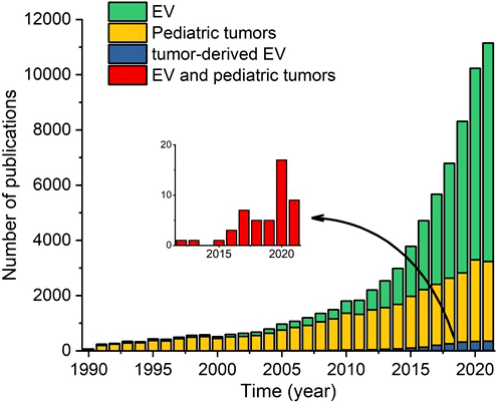
Number of papers published in the indicated years regarding extracellular vesicles (EVs), pediatric solid tumors, tumor-derived EVs, and both EVs and pediatric solid tumors. The inset shows only the publications regarding both EVs and pediatric solid tumors.

To date, relatively few studies examined the clinical relevance of EVs in pediatric solid tumors, despite the high potential of using liquid biopsies in pediatric patients. To illustrate this research gap, we counted the number of articles published since 1990 involving EVs, pediatric solid tumors, tumor-derived EVs, and EVs in pediatric solid tumors; the results are shown in **Figure 2**. Over the past decade, the number of publications regarding EVs and tumor-derived EVs (in adult cancer) has increased exponentially, and publications regarding pediatric solid tumors also increased, albeit gradually; strikingly, however, the number of publications regarding EVs in pediatric solid tumors has remained extremely low.

In this review, we critically assessed the published *in vivo* and *in vitro* studies involving EVs in pediatric solid tumors, and we discuss the barriers that must be overcome in order to bring EVs from the bench to the pediatric bedside. We focused primarily on studies that report patient-derived EVs, and we examined whether the conclusions drawn from these studies were supported by *in vitro* data. Given the importance of studying EVs using standardized methods with respect to reproducibility, we also evaluated the methods used to isolate and characterize EVs, and we assessed whether validation studies using either patient cohorts or *in vitro* methods were reported.

## Methodology

### Search Strategy

**Figure 3:**
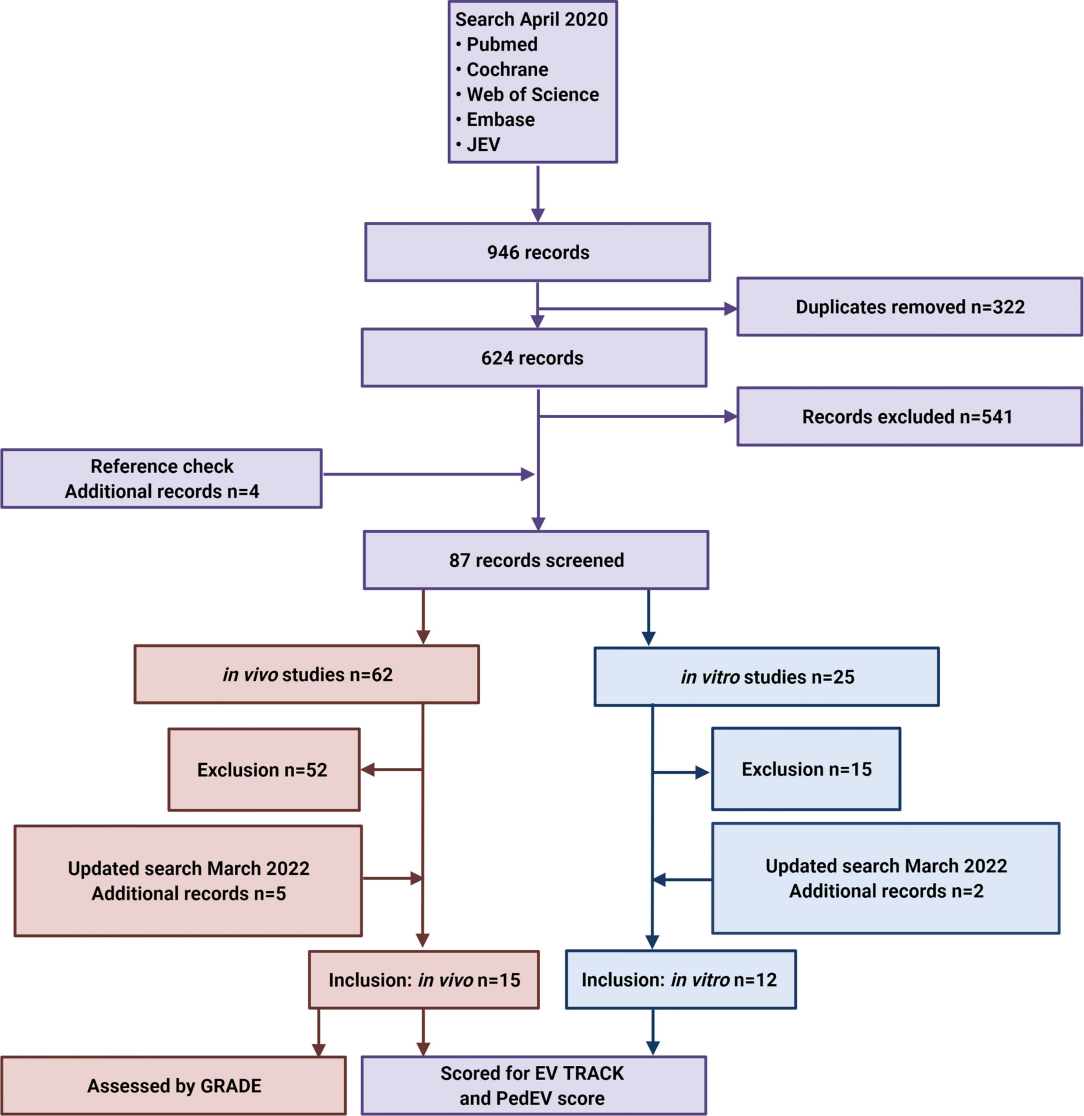
Flow diagram depicting the search strategy and inclusion and exclusion of studies. JEV, Journal of Extracellular Vesicles.

The literature search and review strategy is depicted in **Figure 3**. In brief, we performed an electronic search of the PubMed, Cochrane Library, Web of Science, and Embase databases, as well as the *Journal of Extracellular Vesicles* (*JEV*) website, using the following search terms:

“(“extracellular vesicle” OR “extracellular vesicles” OR EV OR EVs OR exosom* OR ectosom* OR oncosom* OR microvesicle* OR microparticle* OR nanosom* OR nanoparticle* OR “shedding vesicles” OR “exosome-like vesicles”) AND (pediatric OR child OR children OR infant) AND (neuroblastoma OR rhabdomyosarcoma OR sarcoma OR “rhabdoid tumor*” OR “rhabdoid tumor*” OR Wilms OR nephroblastoma OR “renal medullary carcinoma” OR “renal cell carcinoma” OR “renal tumor*” OR leiomyosarcoma OR osteosarcoma OR hepatoblastoma OR “hepatocellular carcinoma” OR “Ewing”)”

Additional eligible studies were identified by screening the references listed in relevant reviews. The final search was performed on April 28, 2020, and EndNote X9 was used to identify and remove duplicate records. We updated the search on March 16^th^ 2022. After pre-screening by two independent investigators (authors EK and NL) based on the title and abstract, followed by subsequent full text screening, a total of 27 studies (15 *in vivo* studies and 12 *in vitro* studies) were included in the final analysis.

### Study Selection

The literature was searched for studies that investigated the use of EVs as a biomarker of pediatric solid tumors. Because we were interested primarily in the clinical relevance of EVs in children with solid tumors, the starting point of our search was *in vivo* studies involving pediatric patients. We then identified *in vitro* studies that investigated the same tumors and included the same authors and/or used the same downstream analysis platform to identify potential biomarkers. Using this approach, we were able to compare studies and investigate whether the *in vitro* data supported the *in vivo* findings. For the *in vivo* part of this review, we included clinical studies that used EVs derived from patients ≤25 years old with pediatric solid tumors. For the *in vitro* part of this review, we included studies that: *i*) assessed EVs from cell lines derived from the same tumor entities as the *in vivo* studies, and *ii*) either used the same platform as the *in vivo* studies or were performed by the same research group as the *in vivo* studies. Only primary reports of original studies were included, and we excluded studies that were published in non-peer-reviewed form such as conference abstracts.

### Grading of Studies

We graded the studies using three approaches. First, we assessed the quality of the clinical studies using the GRADE (Grading of Recommendations, Assessment, Development, and Evaluations) system (**Supplemental Table S1**) (62, 63). Second, we assessed all selected publications (both *in vivo* and *in vitro* studies) by importing all methodological details from these studies into EV-TRACK (https://evtrack.org) in order to obtain their corresponding EV-METRIC scores (50). Although scoring *via* EV-TRACK is highly rigorous and detailed, studies involving pediatric patients are challenging due to the relatively limited volumes of peripheral blood available, which limits the number of techniques that can be applied. Therefore, we also developed a PedEV score. Based on the MISEV guidelines and EV-TRACK score, we defined 11 criteria that are essential to improve reproducibility in pediatric EV studies and included these criteria in our PedEV score (**Supplemental Table S2**). The difference between PedEV and EV-TRACK lies primarily in the score allocated for the EV characterization technique, with PedEV providing a more lenient scoring system of EV characterization compared to EV-TRACK. Data for the evaluation were retrieved from the Materials and Methods sections of the included articles and from the supplementary materials. The 22 publications included in our review are listed in **Table 1**, including each publication’s unique EV-TRACK ID number.

**Table 1:** Critical appraisal of the EV isolation and characterization in the *in vivo* and *in vitro* studies using the criteria for PedEV score and EV-TRACK.

Reference	Nomenclature	Preanalytical variables	Isolation method	Source volume & EV abundance	EV-enriched proteins	Non-EV-enriched proteins	Antibody & lysis buffer	Single vesicle characterisation	Electron microscopy	Characterisation platform	Inclusion of controls	PedEV Score (%)	EV Track ID	EV-METRIC *in vivo* (%)	EV-METRIC *in vitro* (%)
Colletti 2019 (64)	0	5.5	11	11	0	0	NA	11	5.5	11	0	55	EV200162	17	–
Liu 2016 (65)	0	5.5	11	0	0	0	NA	0	0	11	0	27.5	EV200163	0	–
Jiao 2017 (66)	0	5.5	11	0	0	0	NA	0	0	11	0	27.5	EV200164	0	–
Ma 2019 (67)	5.5	5.5	11	5.5*	5.5*	5.5*	5.5*	11*	5.5*	11*	0	71.5	EV200165	38	0
Morini 2019 (68)	5.5	5.5	11	5.5	5.5	0	5.5	5.5	0	11	0	55	EV200166	0	–
Challagundla 2015 (79)	0	5.5	11	5.5	5.5	0	11	5.5	0	11	5.5	60.5	EV210115	–	0
Haug 2015 (80)	5.5	11	11	0	11	5.5	11	11	5.5	11	5.5	88	EV210117	–	44
Xu 2017 (69)	0	0	5.5**	0	0	0	NA	0	0	5.5	0	11	EV210073	0	–
Baglio 2017 (70)	5.5	5.5	11	5.5	5.5*	0	11*	5.5*	5.5*	11	0	66	EV210074	0	22
Shen 2016 (71)	0	5.5	11	0	5.5	0	11	5.5	5.5	5.5	0	49.5	EV210080	25	–
Gong 2018 (72)	0	5.5*	11	0	11*	5.5*	11*	11*	5.5*	5.5	0	66	EV210072	0	44
Jerez 2017 (81)	5.5	5.5	11	0	11	0	11	5.5	5.5	11	5.5	71.5	EV210071	–	22
Jerez 2019 (85)	5.5	5.5	11	5.5	11	0	11	5.5	0	11	5.5	71.5	EV210070	–	14
Fujiwara 2017 (83)	0	5.5	11	0	0	0	NA	0	0	11	0	27.5	EV210116	–	0
Yoshida 2018 (82)	0	5.5	11	0	5.5	0	11	11	5.5	11	5.5	66	EV210079	–	22
Macklin 2016 (84)	5.5	11	11	5.5	11	0	11	11	5.5	11	0	82.5	EV210078	–	38
Raimondi 2019 (86)	5.5	11	5.5	0	5.5	5.5	11	5.5	0	11	0	60.5	EV210081	–	44
Ye 2020 (73)	0	5.5	11	5.5	11*	0	5.5	5.5*	5.5*	11	5.5	66	EV220086	11	0
Cambier 2021 (74)	5.5	11	5.5	5.5	0	0	0	5.5	0	11	11	55	EV220085	29	–
Ghayad 2016 (87)	0	5.5	11	5.5	11	5.5	11	5.5	5.5	11	0	71.5	EV210077	–	33
Rammal 2019 (88)	0	5.5	11	0	11	5.5	11	5.5	5.5	11	0	66	EV210082	–	33
Ghamloush 2019 (75)	0	5.5	5.5	5.5	11*	5.5*	11*	5.5*	5.5*	11	0	66	EV200167	0	38
Miller, 2013 (89)	5.5	5.5	11	11	5.5	5.5	0	11	5.5	11	5.5	77	EV130146	–	25
Zhang 2018 (90)	5.5	5.5	11	5.5	0	0	0	11	5.5	11	5.5	60.5	EV220168	–	29
Dong 2020 (76)	0	5.5	11	5.5	5.5	0	0	5.5	5.5	11	5.5	55	EV220170	0	0
Samuel 2020 (77)	5.5	5.5	11	5.5	5.5	0	0	5.5	0	11	11	60.5	EV220169	0	11
Sun 2022 (78)	5.5	11	11	5.5	0	0	0	5.5	5.5	11	5.5	60.5	EV220167	0	0

See **Supplementary Table S2** for a detailed description of the used criteria. *: performed either in vitro or in vivo, but not both. **: in the Materials and Methods section, EV isolation from conditioned media is mentioned, but from the rest of the article it is clear that this should be serum.

If different EV-METRIC scores were given to different experiments, only the highest score is reported.

## Results and Discussion

### Literature Search

The initial literature search yielded 241 papers in PubMed, 2 papers in the Cochrane Library, 160 papers in Web of Science, 515 papers in Embase, and 28 papers published in the *Journal of Extracellular Vesicles* (**Figure 3**). After duplicates were removed, pre-screening of the remaining 652 articles led to the exclusion of an additional 541 articles due to a lack of relevance. An additional 4 papers were then identified by checking the reference lists. The full text articles describing 62 *in vivo* studies and 30 *in vitro* studies were then assessed for the inclusion and exclusion criteria, and on 16^th^ of March 2022 the search was updated. Finally, this resulted in the inclusion of 15 *in vivo* studies (7 only *in vivo* experiments and 8 both *in vivo* and *in vitro* experiments) and 12 fully *in vitro* studies. We found publications describing six tumor entities (desmoplastic small round cell tumor, hepatoblastoma, neuroblastoma, osteosarcoma, rhabdomyosarcoma and Ewing sarcoma); no other pediatric solid tumors were described.

### Extracellular Vesicles in Pediatric Solid Tumors

The *in vivo* and *in vitro* studies are summarized in **Tables 2**, **3**, respectively. Regarding the *in vivo* studies, we reviewed the following information: tumor type, the sample used to detect EVs, and the sample volume, the latter of which is particularly important in pediatric patients, as sample volumes are typically relatively low. To assess the possible effects of specific EV enrichment techniques on the results, we also examined the enrichment protocols used in each study. We also noted any details regarding the patient cohorts and—if included in the study—healthy controls. As an outcome, we examined the biomarkers, including their function and how this was determined in the study.

**Table 2:** Overview of *in vivo* studies involving EVs derived from pediatric solid tumors.

Tumor type	EV source	Method	Cohort		Result	Biological function
Author reference	Starting amount	Isolation Platform	Patients: Test cohort Validation cohort	Healthy controls		
**Desmoplastic small round cell tumor**				
Colletti (64)	Plasma	Precipitation (miRCURY Exosome Serum/Plasma Kit)	Test cohort: DSRCT n=3 (3 metastatic)	HC n=4	miRNA	Cell growth, proliferation, migration, invasion 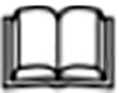
	0.6 mL	Exiqon miRNA PCR panel (175 targets)	Time: diagnosis (n=1), disease progression (n=2)		↑ miR-34a-5p	
			Validation cohort: NR		↑ miR-22-3p	
					↑ miR-324-5p	
					↓ miR-150-5p	
					↓ miR-342-3p	
**Hepatoblastoma**						
Liu (65)	Serum NR	Precipitation (ExoQuick) TaqMan miRNA assay (target: miR-21)	Test cohort: HB n=32 (8 metastatic, 24 localised) Stage: I (n=3), II (n=5), III (n=10), IV (n=14) Validation cohort: NR	HC n=32	miRNA ↑ miR-21	NR
Jiao (66)	Serum NR	Precipitation (ExoQuick) TaqMan miRNA assay (targets: miR-34a, miR-34b, miR-34c)	Test cohort: HB n=63 (14 metastatic, 49 localised) Stage: I (n=7), II (n=10), III (n=20), IV (n=26) Validation cohort: HB n=26 (7 metastatic, 19 localised) Stage: I (n=2), II (n=2), III (n=9), IV (n=13)	HC n=63	miRNA ↓ miR-34a ↓ miR-34b ↓ miR-34c	Tumor initiation, metastasis, progression 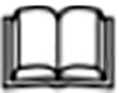
**Neuroblastoma**						
Ma (67)	Plasma 2 mL	Membrane-based affinity binding (exoRNeasy Serum/Plasma Midi Kit) BGIseq-500 miRNA platform (500 targets)	Test cohort: NBL n=9, GNBi n=6 (12 FH, 3 UFH) INSS stage: I (n=2), II (n=4), III (n=5), IV (n=4) Validation cohort: NBL n=8 (6 FH, 2 UFH) INSS stage: I (n=1), II (n=3), III (n=2), IV (n=2)	HC n=7	miRNA ↑ miR-199a-3p	Cell proliferation, migration 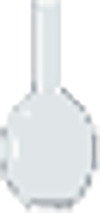
Morini (68)	Plasma 0.5 mL	Membrane-based affinity binding (exoRNeasy Serum/Plasma Midi Kit) TaqMan miRNA array (381 targets)	Test cohort: NB n=52 Time: before + after induction chemotherapy INSS stage: IV (n=47), III (n=4), IVS (n=1) Validation cohort: NR		miRNA ↓ miR-29c ↓ miR-342-3p ↓ let-7b	Response to induction 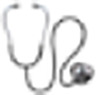 Tumor progression, chemoresistance, survival 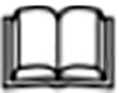
**Osteosarcoma**						
Xu (69)	Serum NR	Differential centrifugation (10 min 1,000 g, 10 min 2,000 g, 30 min 10,000 g, 2 x 70 min 100,000 g) TaqMan miRNA array (746 targets)	Test cohort: OS n=28 (poor response), OS n=25 (good response) Validation cohort: OS n=20 (poor response), OS n=20 (good response)	Test cohort: HC n=31 Validation cohort: HC n=20	miRNA ↑ miR-135b ↑ miR-148a ↑ miR-27a ↑ miR-9 ↓ miR-124 ↓ miR-133a ↓ miR-199a-3p ↓ miR-385	Response to chemotherapy 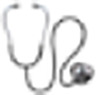 Proliferation, invasion, migration, tumor progression 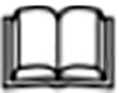
		Differential centrifugation (10 min 1,000 g, 10 min 2,000 g, 30 min 10,000 g, 2 x 70 min 100,000 g) TaqMan mRNA assay (8 targets)	Test cohort: OS n=20 (poor response) OS n=20 (good response) Validation cohort: NR	Test cohort: HC n=20 Validation cohort: NR	mRNA ↑ Annexin2 ↑ Smad2 ↑ Cdc5L ↑ P27 ↓ MTAP ↓ CIP4 ↓ PEDF ↓ WWOX	Response to chemotherapy 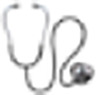
Baglio (70)	Serum 1.5 mL	Size exclusion chromatography ELISA (target: TGFβ)	Test cohort: OS n=10 Stage: IB (n=4), IIA (n=2), IIB (n=2), III (n=2) Validation cohort: NR	HC n=10	Protein ↑ TGFβ	Tumor growth, metastasis 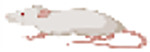
Shen (71)	Serum NR	Precipitation (ExoQuick) Western blotting (target: G6PD)	Test cohort: OS n=15 Time: diagnosis Validation cohort: NR	HC n=15	Protein ↑ G6PD	Cell adhesion, migration, viability 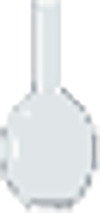
Gong (72)	Plasma NR	Differential centrifugation (10 min 300 g, 10 min 2,000 g, 30 min 10,000 g, 2x 70 min 100,000 g) Small RNA library sequencing (Illumina)	Test cohort: OS n=2 (localised) Time: diagnosis + postoperative metastasis Validation cohort: OS n=3 (lung metastasis), OS n=3 (localised) Time: diagnosis		miRNA ↑ miR-675	Migration, invasion 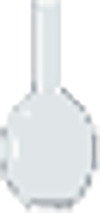 Metastasis 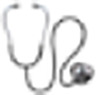
Ye (73)	Plasma NR	Differential centrifugation (20 min 1,500G, 30 min 10,000G, 120 min 100,000G) Small RNA sequencing (BGISEQ-500) RT-qPCR	Test cohort: OS n=25 Validation cohort: NR	HC n=10	miRNA ↑miR92a-3p ↑miR130a-3p ↑miR195-3p ↑miR335-5 ↑let7i-3p	Proliferation, apoptosis inhibition, G2/M cell cycle arrest, invasion 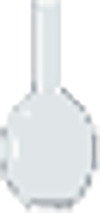
Cambier (74)	Serum 0.3ml	Precipitation (Exoquick) Precipitation (PEG) Size exclusion chromatography Immunoaffinity capture	Test cohort: OS n=12 Validation cohort: OS n=8	HC n=12 HC n=12	DNA ↑HSATII ↑LINE1-P1 ↑Charlie3 RNA =HSATII =LINE1-P1 =Charlie3	NR
**Rhabdomyosarcoma**						
Ghamloush (75)	Serum 0.4 mL	Differential centrifugation (10 min 300 g, 10 min 2,000 g, 30 min 10,000 g, 2x 70 min 100,000 g) + precipitation (ExoQuick) TaqMan miRNA assay (target: miR-486)	Test cohort: RMS n=7 (ERMS n=6, ARMS n=1), control n=6 (benign tumor) Time: diagnosis Follow-up n=2 (ERMS n=1, ARMS n=1) Time: follow-up after treatment		miRNA ↑ miR-486-5p	Response to chemotherapy in ARMS 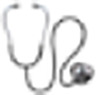 Invasion, migration, proliferation 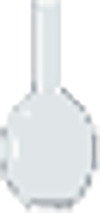
**Ewing sarcoma**						
Dong (76)	Plasma 0.3 mL	ES-EV Click Chip RT-ddPCR	Test cohort: ES n=4 Time: NR	HC=4	mRNA EWSR1 rearrangement	NR
Samuel (77)	Plasma 0.25 mL	Immunoprecipitation qRT-PCR	Test cohort: ES n=10	HC=6	mRNA EWSR1-ETS fusion	NR
Sun (78)	Plasma 1.0 mL	Click Beads RT-dPCR	Test cohort: ES n=28 (35 patients)	HC=10	mRNA EWSR1-FLI1	NR

DSRCT, desmoplastic small round cell tumor; HC, healthy control; HB, hepatoblastoma; NR, not reported; PRETEXT, pre-treatment extent of disease; NBL, neuroblastoma; GNBi, ganglioneuroblastoma intermixed; FH, favourable histology; UFH, unfavourable histology; INSS, International Neuroblastoma Staging System; OS, osteosarcoma; ERMS, embryonal rhabdomyosarcoma; ARMS, alveolar rhabdomyosarcoma; RMS, rhabdomyosarcoma. Function derived from: 
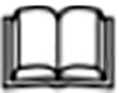
 : literature; 
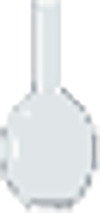
 : in vitro; : 
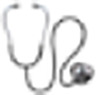
 clinical; 
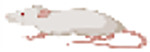
 : mice.

**Table 3:** Overview of the *in vitro* studies involving pediatric solid tumors and EVs.

Tumor type	Cell lines	Method	Result	Biological function
		*Isolation Platform*		
**Neuroblastoma**				
Ma (67)	SK-N-SH SH-SY5Y SK-N- BE(2)	Differential centrifugation (10 min 300 g, 10 min 2,000 g, 30 min 10,000 g, 70 min 100,000 g, 60 min 100,000 g) BGIseq-500 miRNA platform (500 targets)	miRNA ↑ miR-199a-3p	Cell proliferation, migration 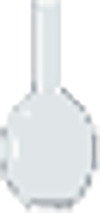
Challagundla (79)	SK-N-BE(2) CHLA-255 IMR-32	Precipitation (ExoQuick) Affymetrix human exon arrays (> 10^6^ targets)	miRNA ↑ miR-21-5p	Drug resistance 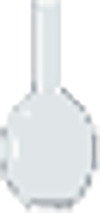
Haug (80)	MYCN-amplified Kelly MYCN-amplified SK-N-BE(2)-C SK-N-AS	Differential centrifugation (10 min 200 g, 20 min 2,000 g, 30 min 10,000 g, 70 min 110,000 g) miRCURY qPCR panels 1 + 2 V2.M (752 targets)	miRNA ↑ miR-92a-3p ↑ miR-23a-3p ↑ miR-218-5p ↑ miR-320a ↑ miR-24-3p ↑ miR-27b-3p ↑ miR-16-5p ↑ miR-25-3p ↑ miR-21-5p ↑ miR-125b-5p ↑ miR-320b	Survival, proliferation, apoptosis, angiogenesis, differentiation, invasion, metastasis 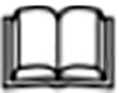
**Osteosarcoma**				
Baglio (70)	MG63 HOS 143B	Differential centrifugation (2x 10 min 500 g, 2x 15 min 2,000 g, 2x 30 min 10,000 g, 2x 60 min 70,000 g) ELISA (target: TGFβ)	Protein ↑ TGFβ	Tumor growth, metastasis 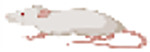
Gong (72)	MG63 HOS 143B Well5	Differential centrifugation (10 min 300 g, 10 min 2,000 g, 30 min 10,000 g, 2x 70 min 100,000 g) Small RNA library sequencing (Illumina)	miRNA ↑ miR-675	Migration, invasion 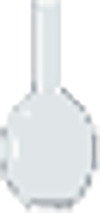 Metastasis 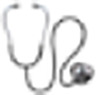
Jerez (81)	SAOS2 MG63 U2OS HOS 143B	Ultracentrifugation (90 min 100,000 g) Proteomics (MS)	Protein 565 unique proteins	Angiogenesis, adhesion, migration, metastasis 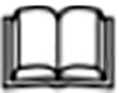
Yoshida (82)	143B U2OS	Ultracentrifugation (2x 70 min 110,000 g) RT-qPCR (target miR-25-3p)	miRNA ↑ miR-25-3p	Proliferation, invasion, migration, angiogenesis, drug resistance 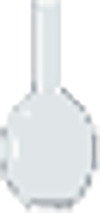
Fujiwara (83)	U2OS HOS 143B SaOS2	Ultracentrifugation (70 min 110,000 g) RT-qPCR (target miR-25-3p)	miRNA ↑ miR-25-3p ↑ miR-17-5p	Cell proliferation, tumor growth 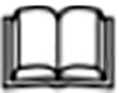 Survival 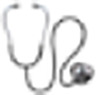
Macklin (84)	KHOS (HiMet-C1, HiMet-C6, LoMet-C4, LoMet-C5)	Precipitation (ExoQuick) Proteomics (MS)	Protein 31 unique proteins	Migration, invasion 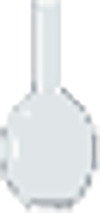 Lung metastasis 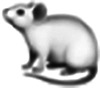
Jerez (85)	SAOS2 MG63 HOS 143B U2OS hFOB1.19	Ultracentrifugation (90 min 100,000 g) NEBNext Small RNA library (Illumina)	miRNA ↑ miR-21-5p ↑ miR-143-3p ↑ miR-181a-5p ↑ miR-148a-3p	Tumor progression, metastasis 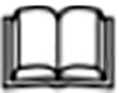
Raimondi (86)	SAOS2 MG63 U2	Differential centrifugation (5 min 300 g, 15 min 3,000 g, 30 min, 10,000 g, 90 min 100,000 g) MiSeq Reagent Kit v3 (Illumina)	miRNA ↑ miR-21-5p ↑ miR-148a-3p	Carcinogenesis 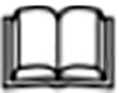
Ye (73)	NHOst U2OS 143B	EV isolation not reported RT-qPCR	miRNA miR130a-30 miR195-3p	Proliferation, apoptosis inhibition, G2/M cell cycle arrest, invasion 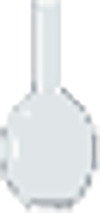
**Rhabdomyosarcoma**				
Ghayad (87)	Rh30 Rh41 RD JR1 Rh36	Differential centrifugation (10 min 300 g, 10 min 2,000 g, 30 min 10,000 g, 2x 70 min 100,000 g) Affymetrix GeneChip miRNA 3.0 Arrays kit (19724 targets)	miRNA ↑ miR-1246 ↑ miR-1268	Proliferation, migration, invasion, metastasis 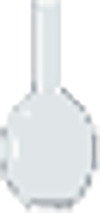
Rammal (88)	Rh30 Rh41 RD JR1 Rh36	Differential centrifugation (10 min 300 g, 20 min 2,000 g, 30 min 10,000 g, 2x 70 min 100,000 g) Proteomics (MS)	Protein 36 unique proteins	Invasion, proliferation, metastasis 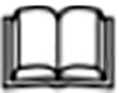
Ghamloush (75)	Rh30 Rh41 RD JR1 Rh36	Differential centrifugation (10 min 300 g, 10 min 2,000 g, 30 min 10,000 g, 2x 70 min 100,000 g) TaqMan miRNA assay (target: miR-486)	miRNA ↑ miR-486-5p	Invasion, migration, proliferation 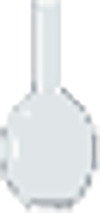
**Ewing sarcoma**				
Miller (89)	A673 SK-N-MC SB-KMS-KS1	Differential centrifugation 10 min 300 g, 10 min 2,000g, 30 min 10,000, 70 min 100,000, 60 min 100,000 Affymetrix HumanGene 1.0 ST arrays	mRNA NR0B1, NKX2.2, STEAP1, LIPI, EWSR1-FLI fusion	Signal transduction, stemness 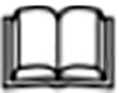
Zhang (90)	Hs919.T CHLA-258 CHLA-9	Differential centrifugation 5 min 2500 rpm, 45 min 10,000 g, 120 min 100,000 g	mRNA EWSR1-FLI1 fusion	NR
Dong (76)	A673 SK ES 1 ES5838	Differential centrifugation (10 min 300 g, 30 min 4600 g, 120 min 100,000 g) Exoquick Immunomagnetic beads ES-EV Click Chips RT-ddPCR	mRNA EWSR1 rearrangement	NR
Samuel (77)	TC-71 RD-ES SK-ES-1 CHLA-258 COG-E-352 Hs919.T	Differential centrifugation 5 min 2500 rpm, 45 min 10,000 g, 75 min 110,000 g, 60 min 35,800 rpm Proteomics Immunoprecipitation RT-qPCR	Protein Bulk analysis CD99, NGFR mRNA EWSR1-ETS fusion	Bulk: Exosomal proteins (membrane transport and fusion), metabolic enzymes, antigen presenting, cytoskeletal, protein binding 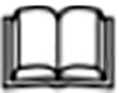
Sun (78)	A673	Differential centrifugation 10 min 300 g, 10 min 2800 g, 90 min 100,000 g Click beads ExoQuick Magnetic biotin-PEG-DSPE beads RT-dPCR	mRNA EWSR1-FLI1 fusion	NR

MS, mass spectrometry. Function derived from: 
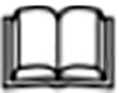
 : literature; 
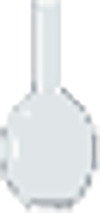
 : in vitro; 
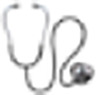
 : clinical; 
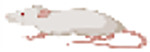
 : mice.

Next, we critically assessed the clinical studies using the GRADE system (62, 63) and the EV methodology using our own PedEV score and EV-TRACK score (51) (**Table 1**, **4**). The mean GRADE score was 7.7 points (range: 5-11 points), and the mean PedEV score was 59.1% (range: 11-88%). Finally, the mean EV-TRACK score was 8% (range: 0-38%) for the *in vivo* studies and 21% (range: 0-44%) for the *in vitro* studies. Below, we discuss the output for each of the six tumor entities.

**Table 4:** Critical appraisal of the clinical studies using the GRADE system.

Reference	Study design	Patient inclusion	Patient characteristics	Selection bias	Reproducibility	*In vitro* validation	End point	Outcome	Funding	Score
Colletti (64)	2	0	1	0	0	0	1	2	1	7
Liu (65)	2	1	1	0	0	0	1	2	1	8
Jiao (66)	1	1	1	0	1	0	1	2	1	8
Ma (67)	2	1	1	0	2	1	1	2	1	11
Morini (68)	2	1	1	0	0	0	1	2	1	8
Xu (69)	2	1	1	0	2	0	1	2	1	10
Baglio (70)	2	0	1	0	0	1*	1	2	0	7
Shen (71)	2	1	1	0	0	0	1	2	1	8
Gong (72)	2	1	0	0	2	1*	1	2	1	10
Ye (73)	2	0	1	1	0	1	1	1	0	7
Cambier (74)	2	0	1	0	1	0	1	2	0	7
Ghamloush (75)	1	0	0	0	0	1*	1	2	0	5
Dong ( (76)	1	0	0	0	0	2	1	0	1	5
Samuel ( (77)	1	1	0	1	0	1	1	1	1	7
Sun (78)	1	1	1	1	0	1	1	2	0	8

See **Supplementary Table S1** for a detailed description of the criteria. *: in vivo validation of in vitro findings.

#### Desmoplastic Small Round Cell Tumor

Desmoplastic small round cell tumor (DSRCT) is an aggressive and rare sarcoma that occurs primarily in adolescents and young adults, with an increased prevalence among males (91). The majority of DSRCT cases present intra-abdominally, often with widespread metastasis throughout the abdomen (92). At the molecular level, DSRCT is characterized by a t(11:22)(p13;q12) translocation, causing fusion of the *EWSR1* and *WT1* genes (93). The resulting fusion gene generates the oncogenic EWSR1-WT1 fusion protein, which regulates transcriptional activity and is essential for tumor cell proliferation (94). Patients with DSRCT have extremely poor outcome, and sparse research has been performed with respect to diagnostic and prognostic biomarkers (95, 96). Our literature search identified only one clinical study involving EV in DSRCT and no *in vitro* studies.

Colletti et al. examined the miRNA profiles of EVs isolated from plasma samples obtained from three patients with DSRCT and compared the results with EVs obtained from four healthy controls (**Table 2**) (64). They found that five miRNAs were highly dysregulated in all three patients, and the dysregulated miRNAs were correlated with both tumor aggressiveness and clinical outcome, suggesting that this EV-derived miRNA profile could be used as a possible prognostic marker. Moreover, bioinformatics analysis showed that the genes targeted by the dysregulated miRNAs are involved in oncogenic signaling pathways. A potential limitation of this study is that the authors reported, using western blot analysis, to detect EV-related and non-EV-related proteins, but did not show the results of these experiments. Other methodological limitations include the relatively small cohort size (with 3 patients and 4 controls), no clear list of inclusion and exclusion criteria, and no validation in an independent cohort, which complicates the translation to clinical practice. These limitations are reflected in the relatively low GRADE and EV-TRACK scores of 7 and 17%, respectively, although the PedEV score (55%) was average, indicating a more permissive assessment of their EV characterization.

Given that DSRCT is extremely rare, validation in an independent cohort may be difficult. However, *in vitro* validation of the results would likely increase their applicability and provide important insights into the pathology underlying DSRCT.

#### Hepatoblastoma

Hepatoblastoma is the most common primary pediatric liver tumor, typically presenting in children between 6 months and 4 years of age (97). Hepatoblastoma is an embryonal tumor, presumably arising from hepatocyte precursor cells and displaying histological patterns that recapitulate the liver’s developmental stages (98). Although most hepatoblastoma cases are sporadic in origin, some are associated with genetic syndromes such as Beckwith-Wiedemann syndrome or familial adenomatous polyposis (99). In recent decades, the overall survival rate among patients with hepatoblastoma has improved considerably; however, the outcome for patients with advanced disease remains unfavorable, and effective biomarkers for early diagnosis and for predicting outcome are still lacking (100). Our literature search revealed two clinical studies regarding EV in hepatoblastoma, and no *in vitro* studies.

Liu et al. examined the diagnostic and prognostic potential of measuring miR-21 in serum EVs in patients with hepatoblastoma (**Table 3**) (65). The authors found significantly higher expression of miR-21 in both the serum and serum-derived EVs in patients compared to healthy controls. They also showed that miR-21 expression in EVs is a better diagnostic marker for hepatoblastoma than serum AFP (alpha-fetoprotein) levels, the currently used biomarker (101). miR-21 expression was also found to be an independent predictor of low event-free survival, suggesting that it could be used as both a diagnostic and prognostic biomarker for hepatoblastoma. Although they did not assess the function of miR-21 in hepatoblastoma, the authors noted that this will be examined in a follow-up study. In addition, future studies are needed in order to determine the precise prognostic value of miR-21, as well as the relationship between this marker and other risk factors, which may confer a possible bias. Finally, the size of their study cohort (n=32 patients) was relatively large given the rarity of this tumor, and the authors included a control group consisting of healthy age- and gender-matched children; nevertheless, a validation cohort and/or *in vitro* validation is needed in order to support their conclusions.

Jiao et al. studied the diagnostic and prognostic value of measuring miR-34 expression in serum-derived EVs in patients with hepatoblastoma (**Table 3**) and found lower levels of miR-34a, miR-34b, and miR-34c in EV-enriched samples obtained from patients compared to healthy age- and gender-matched controls (66). With respect to diagnosing hepatoblastoma, they found that a panel comprised of all three miRNAs performed better than serum AFP levels, indicating its potential as a diagnostic biomarker. Moreover, this miRNA panel appeared to be superior at predicting poor prognosis compared to other risk factors. The authors also reported that miR-34 miRNAs have been shown previously to play a role in the initiation, progression, and metastasis of several types of tumors. Although the authors did not investigate the function of miR-34 miRNAs specifically in hepatoblastoma, their study included a relatively large patient cohort (n=63) and an age- and gender-matched control group; moreover, they also included a validation cohort (n=26 patients). On the other hand, a potential limitation of their study is that it was retrospective.

Remarkably, although the studies by Liu et al. (65) and Jiao et al. (66) were performed by two different groups at two different research centers, their publications contained large sections of identical text (particularly their description of the methods), and the studies were performed during the same time period with comparable cohorts. In addition, although the two groups used a similar approach, they studied different miRNAs, without discussing their choice of miRNAs.

An important limitation common to both studies is a general lack of EV characterization. Furthermore, they provided no evidence that the miRNAs were EV-associated, nor did they report the initial volume of serum. These limitations are reflected in the low EV-METRIC and PedEV scores (0% and 27.5%, respectively, for both studies), although their GRADE score of 8 was average.

#### Neuroblastoma

Neuroblastoma is the most common pediatric extracranial solid tumor, predominantly occurring in children in the first 2 years of life (102). Neuroblastoma arises from the developing sympathetic nervous system, resulting in tumors in the adrenal glands and/or sympathetic ganglia. Neuroblastoma is characterized by biological heterogeneity and unique clinical properties such as a tendency for spontaneous regression in infants, even in cases with metastatic disease (103). These features translate to a highly variable outcome, with a survival rate higher than 90% in low-risk and intermediate-risk cases, but only 40-50% survival in high-risk cases (104). Several genetic aberrations have been associated with neuroblastoma, including mutations in the *ALK* (105) and *PHOX2B* (106) genes, amplification of the *MYCN* gene (107), and segmental chromosome alterations (108). Importantly, new biomarkers for the early detection of neuroblastoma and for predicting the patient’s response to therapy are urgently needed. With respect to EVs in neuroblastoma, our literature search revealed two clinical studies regarding EVs in neuroblastoma (one of which also assessed EVs *in vitro*) and two *in vitro* studies.

Ma et al. identified EV-derived miRNA biomarkers *in vivo* and then examined the underlying molecular mechanism in an *in vitro* study (**Tables 2**, **3**) (67). In their *in vivo* study, they used next-generation sequencing of EV-derived miRNA and found that the expression of miR-199a-3p was significantly higher in EVs isolated from plasma obtained at the initial diagnosis of patients with neuroblastoma (in all risk groups) compared to healthy age- and gender-matched controls. Moreover, this upregulation of miR-199a-3p in patients appeared to be correlated with a high risk profile. In their *in vitro* study, the authors found that miR-199a-3p was expressed at significantly higher levels in neuroblastoma cell lines and their corresponding EVs compared to control human cell lines, including HUVEC (human umbilical vein endothelial cells), HEK293, and MRC-5 (fibroblast) cells. This miRNA was also shown to promote the proliferation and migration of neuroblastoma cells. Based on their results, the authors suggest that miR-199a-3p may be used as a rapid, easy, non-invasive biomarker for the detection of neuroblastoma, even though their study included only 7 healthy controls. With respect to the authors’ *in vitro* validation of their *in vivo* findings, it is important to note that they used different methods to isolate EVs, and only the patient-derived EVs were characterized. Moreover, their *in vivo* study had a relatively small cohort (n=15 patients) and was cross-sectional; thus, longitudinal studies involving several time points and larger cohorts may provide more insights into the progression of neuroblastoma and facilitate the discovery of new biomarkers. Nevertheless, their validation using both a clinical validation cohort (n=8) and *in vitro* data increase their study’s reproducibility. The resulting GRADE score of 11 indicates that this was a well-balanced study; in addition, the study used a sound methodological approach for the *in vivo* experiments, reflected by the relatively high EV-TRACK and PedEV scores of 38% and 71.5%, respectively.

Morini et al. investigated whether EV-derived miRNA can be used to predict the patient’s response to induction chemotherapy (**Table 2**) (68). The authors found that plasma samples from patients with high-risk neuroblastoma contained significant levels of neuroblastoma-derived EVs, and these levels decreased and developed a differential miRNA expression profile in response to chemotherapy. Specifically, they found that a signature consisting of three miRNAs (miR-29c, miR-342-3p, and let-7b) could discriminate between patients with a poor clinical response and patients with a good clinical response. These three miRNAs have tumor-suppressor functions, and pathway analysis indicated that they play a role in tumor progression, survival, and chemoresistance. Notably, for each patient the authors also calculated a chemoresistance index for the specific drugs used in neuroblastoma treatment, based on changes in EV-derived miRNAs; they found that this index reliably defined each patient’s response to specific drugs, creating new opportunities for applications involving personalized medicine. Despite these strengths, their study was retrospective and lacked *in vivo* and *in vitro* validation. Thus, a prospective study involving a validation cohort would likely support the prognostic value of these miRNAs. Moreover, their characterization of EVs did not use conventional techniques such as western blot analysis or electron microscopy, which resulted in an EV-METRIC score of 0%. In contrast, the PedEV score was 55%; this higher PedEV score was due to their use of flow cytometry to analyze EVs. However, all of the essential information regarding the use of flow cytometry needs to be properly reported to avoid an erroneous interpretation of the data, particularly when analyzing single EV−based flow cytometry data (47).

Challagundla et al. examined the role of EV-derived miRNAs in the development of drug resistance in neuroblastoma (**Table 3**) (79). They measured the expression of several pro-inflammatory miRNAs in three neuroblastoma cell lines and found that only miR-21-5p was expressed in all three cell lines. The authors also claimed that they used a noncoding RNA array to screen for miRNA expression in EVs released by five neuroblastoma cell lines; however, these data were not shown. Co-culture experiments showed that secreted miR-21-5p could be transferred to human monocytes *via* EVs. Thus, although the potential of using miR-21-5p as a biomarker for neuroblastoma was not examined, it would be interesting to analyze whether this miRNA is upregulated *in vivo*. Another interesting question is if miR-21-5p is upregulated only in *MYCN*-amplified neuroblastoma, as the *MYCN* amplification status of the cell lines was not clearly stated. Similar to the study by Morini et al. (68), we found a relatively large discrepancy between the EV-METRIC score (0%) and PedEV score (60.5%). Moreover, the study by Challagundla et al. did not meet the strict criteria established by EV-TRACK, including failing to report an analysis of EV-enriched and non-EV−enriched proteins, and not using a density gradient to purify the EV-enriched fraction. However, the authors did provide details regarding their EV enrichment method, their characterization of EVs using nanoparticle tracking analysis (NTA), and their analysis of the EV cargo, which is reflected in the relatively higher PedEV score (60.5%).

Haug et al. examined the miRNA profile of EVs derived from two *MYCN*-amplified neuroblastoma cell lines (**Table 3**) (80) and found a total of 11 EV-derived miRNAs that were expressed at high levels in both cell lines. Functional enrichment analysis showed that these miRNAs are involved in several processes in cancer, including tumor survival, proliferation, and metastasis. A strength of this study is that they validated the origin of the isolated miRNAs by measuring the expression of EV-derived miRNAs in a single neuroblastoma cell line using two different isolation protocols, yielding nearly identical expression levels. Among all of the publications that we analyzed, this study had the highest EV-METRIC (44%) and PedEV (88%) scores, reflecting its sound methodology and study design.

Among these four studies, miR-199a-3p was the only miRNA reported to be upregulated in neuroblastoma both *in vivo* and *in vitro* (67). In addition, miR-21-5p was upregulated in two *in vitro* studies (79, 80). Based on the various groups’ reporting of their EV methodologies, we found disparity between the EV-METRIC and PedEV scores. This disparity reflects the efforts that the researchers put into characterizing EVs, but it also reflects possible limitations with respect to EV-specific equipment and/or the knowledge available at the various research centers.

#### Osteosarcoma

Osteosarcoma is a highly aggressive primary bone tumor that typically presents in children and adolescents, although a second peak in incidence can occur among individuals >60 years of age (109). The primary tumors typically arise in the appendicular skeleton, with metastatic disease commonly occurring in the lungs and other bones (110). The tumor is mesenchymal in origin and is characterized by the production of osteoid (111), and includes a wide range of distinct histological subtypes (112). Although the genetic landscape of osteosarcoma varies widely between tumors, osteosarcoma has been associated with recurrent somatic mutations in several genes, including *TP53*, *RB1*, *ARTX*, and *DLG2* (113, 114). The survival rate among patients with metastatic disease remains low, emphasizing the urgent need to identify reliable biomarkers for diagnosis and tracking the disease progression (115). Our search revealed six *in vivo* studies involving EVs in osteosarcoma (of which three studies also included *in vitro* experiments) and six distinct *in vitro* studies.

Xu et al. examined the potential of using serum EV−derived miRNA expression profiles to predict the response to chemotherapy in patients with osteosarcoma (**Table 2**) (69). The authors identified the differential expression of 30 miRNAs, 8 of which were confirmed in a validation cohort, and they found that the expression levels were correlated with poor response. Comparative pathway analysis revealed that the differentially regulated miRNAs affect several pathways involved in cancer. Based on these results, the authors suggest that both miRNAs and mRNAs derived from EVs could be used as markers to monitor and predict disease progression in patients with osteosarcoma undergoing chemotherapy. This study had several strengths, including the use of a uniform method for EV enrichment in all samples, the relatively large size of the patient cohort (n=53) and validation cohort (n=40), and their assessment of both miRNA and mRNA. On the other hand, a limitation of their study is that pre-analysis factors such as the collection and processing of the serum samples were not described, and no results were reported with respect to EV characterization or validation. These limitations are reflected in both a low EV-METRIC score (0%) and a low PedEV score (11%). In contrast, the GRADE score was 10, which is relatively good.

Baglio et al. studied the effect of tumor EV−educated mesenchymal stem cells on osteosarcoma progression (**Tables 2**, **3**) (70). They found that EVs derived from three osteosarcoma cell lines contained higher levels of transforming growth factor β (TGFβ) compared to EVs derived from fibroblast cells (as a control group). They also studied the effect of osteosarcoma-derived EVs on tumor growth and metastasis in a preclinical mouse model. Finally, they measured serum TGFβ levels in osteosarcoma patients and healthy controls and found increased levels in the patient group; however, they did not indicate whether the healthy controls were age-matched. Importantly, this study was not designed to identify biomarkers for osteosarcoma, but rather to perform an *in vitro* analysis of osteosarcoma-derived EVs. Furthermore, they used different EV isolation protocols for the *in vitro* and *in vivo* samples. This difference is reflected in the EV-METRIC scores of 0% and 22% for the *in vivo* and *in vitro* experiments, respectively. This difference between the *in vivo* and *in vitro* protocols cannot be captured by the PedEV score (66%), which scores overall methodological quality. Finally, the GRADE score for this study was 7, as the authors failed to report their patient inclusion criteria and no validation cohort was included.

Shen et al. found that serum-derived EVs obtained from patients with osteosarcoma can affect the adhesion, migration, and viability of MG-63 cells, a human pre-osteoblastic cell line (**Table 2**) (71). They then used mass spectrometry (MS) to identify the proteins in these EVs, finding that 233 proteins were expressed in the osteosarcoma patients but not in healthy (albeit not age- or gender-matched) controls. KEGG (Kyoto Encyclopedia of Genes and Genomes) pathway analysis revealed that these proteins play a role in four pathways that are important for osteosarcoma progression. Interestingly, the protein G6PD (glucose-6-phospate dehydrogenase) was expressed at particularly high levels in the EVs obtained from patients with osteosarcoma and was suggested as a diagnostic and/or therapeutic target in osteosarcoma; however, this finding should be substantiated in a validation cohort. More extensive characterization of the EVs and the inclusion of age- and gender-matched healthy controls would have increased the study’s validity; these limitations resulted in a GRADE score of 8. The PedEV score of 49.5% indicates that the EV characterization was reported in sufficient detail; however, the EV-METRIC score was only 25% based on the authors failing to report EV quantitation and not mentioning whether they purified the EV-enriched fraction using a density gradient.

Gong et al. examined the miRNA profiles of EVs isolated from metastatic osteosarcoma cell lines and non-metastatic osteosarcoma cell lines (**Tables 2**, **3**) (72). Small RNA sequencing identified a total of 61 miRNAs that were differentially expressed in EVs between the metastatic and non-metastatic cell lines, as well as patient serum. miR-675 was the most significantly upregulated miRNA in EVs isolated from the metastatic cell lines, and this result was confirmed both *in vitro* and *in vivo* using RT-qPCR. *In vitro* functional studies indicated that miR-675 can increase tumor cell migration and invasion by targeting expression of the calcium-binding protein CALN1 (Calneuron-1); thus, miR-675 might serve as a valuable mechanism-based prognostic biomarker for osteosarcoma metastasis. A strength of this study is that it included both *in vivo* and *in vitro* data. However, it is limited by the small patient cohort (n=2) and the fact that the patient characteristics are not reported. The GRADE score was therefore 10. A follow-up study with a larger clinical cohort is needed in order to validate these findings. The PedEV and EV-METRIC scores were relatively high for the *in vitro* experiments (66% and 44%, respectively); however, the *in vivo* experiments lacked sufficient EV characterization.

Jerez et al. performed a proteomic analysis of EVs derived from three osteosarcoma cell lines (**Table 3**) (81). The authors identified a total of 1,741 proteins that were unique to the osteosarcoma-derived EVs, 565 of which were found in all three cell lines. Gene Ontology analysis revealed that these proteins are involved in angiogenesis, adhesion, and cell migration.

In a separate, more recent study the same group used next-generation sequencing to characterize the miRNAs in EVs derived from five osteosarcoma cell lines, some of which were included in their previous report (**Table 3**) (85). They found 237 miRNAs that were present exclusively in the osteosarcoma cell lines, and they found that the metastatic cell lines clustered differently than the non-metastatic cell lines. In particular, they found four miRNAs (miR-21-5p, miR-143-3p, miR-181a-5p, and miR-148a-3p) that were enriched in the metastatic SaOS2 cell line. Gene Ontology analysis revealed that the genes targeted by these highly abundant miRNAs in osteosarcoma cell lines are related to tumor progression and metastasis. The EV methodology used in both the 2017 and 2019 studies had rather high standards with respect to EV isolation and characterization, resulting in a PedEV score of 71.5% for both studies. However, in their 2019 paper (85) they did not report the results regarding EV characterization by EV-enriched proteins, resulting in a slightly lower EV-METRIC score for this paper (14%) compared to their previous publication (22%).

Fujiwara et al. screened circulating miRNAs in patient serum samples and in EVs secreted by osteosarcoma cell lines (**Table 3**) (83). They found that miR-25-3p and miR-17-5p were upregulated in the osteosarcoma cell lines and culture media, and the expression of these two miRNAs was even higher in EVs derived from the osteosarcoma cell lines than in the cells themselves. They also found that the serum levels of these miRNAs were higher in patients with osteosarcoma than in healthy controls. Due to the limited volume of serum, miRNAs were isolated only from total serum and not from EV-enriched samples. Moreover, the low EV-METRIC and PedEV scores of 0% and 5, respectively, reflect the limited effort that the authors put into providing a detailed description of their isolation and characterization of EVs.

In a follow-up study by the same group, Yoshida et al. assessed the role of miR-25-3p in osteosarcoma (**Table 3**) (82) and found that high expression levels of miR-25-3p were correlated with poor prognosis. They also performed functional analyses and found that this miRNA is involved in proliferation, invasion, migration, and multi-drug resistance in osteosarcoma cells. The encapsulation of the miRNAs in the lipid vesicles was believed to increase the stability of miR-25-3p and facilitate delivery to the tumor microenvironment, promoting tumor progression. In this follow-up study, the authors included more details regarding their EV methodology and characterization, as reflected by the PedEV and EV-METRIC scores of 66% and 22%, respectively.

Macklin et al. analyzed EVs secreted by both high and low metastatic clonal variants of the KHOS human osteosarcoma cell line (**Table 3**) (84). The authors found that the high metastatic cells secreted three times more EVs than the low metastatic cells, and transfer of these EVs to low metastatic cells induced a migratory and invasive phenotype in those cells. Using MS, they identified 64 proteins in the high metastatic cell−derived EVs, 31 of which were unique to these vesicles. In *in vivo* mouse experiments, they also found that high metastatic EVs preferentially colonized the lung tissue, which is the principal site of metastatic development in osteosarcoma (110). The quality of reporting their EV methodology was high, with EV-METRIC and PedEV scores of 38% and 15%, respectively.

Raimondi et al. performed small RNA sequencing on osteosarcoma-derived EVs and on their parental cells (**Table 3**) (86). The authors found a total of 21 differentially expressed miRNAs, and bioinformatic analysis revealed that these miRNAs are associated with carcinogenesis. In addition, they found that expression of miR-21-5p and miR-148a was increased in cultured osteoclast-like and endothelial cells that were treated with osteosarcoma-derived EVs, promoting osteoclast formation and angiogenesis; this finding confirmed the notion that these miRNAs are transferred from EVs to their target cells, in which they exert functional effects. The PedEV score of 82.5% and EV-METRIC score of 44% reflect the fact that the authors reported more details regarding their EV methodology than the other publications assessed in our review.

Ye et al. also performed small RNA sequencing on EV derived from osteosarcoma patients and healthy controls (73). They identified 10 miRNA that were upregulated in patients. They went on to perform RT-qPCR on a selection of these miRNA and compared that to EV from 3 osteosarcoma cell lines. This comparison found only miR195-3p and miR130a-3p to be upregulated in both patient and cell line-derived EV. They further analyzed the function of miR195-3p in several experiments with an osteosarcoma cell line and mice, from which they concluded that miR195-3p promotes cell proliferation and migration, and inhibits apoptosis. The investigators do not state the exact starting volume for EV isolation from plasma. They also do not report the EV isolation method from the cell lines for the functional experiments, nor if these EVs were analyzed by transmission electron microscopy and/or western blot, as was done for the EVs from plasma. Considering the clinical part of the study, a validation cohort is missing, as is a clear description of patient inclusion criteria. This results in a PedEV score of 66% and an EV-METRIC score of 11%, and a GRADE score of 7.

Cambier et al. analyzed repetitive DNA and RNA elements present in EVs isolated from serum from patients and healthy controls (74). In this report, different EV isolation and purification approaches were used: ExoQuick in the discovery cohort and PEG precipitation, SEC and immunoaffinity capture in different subgroups within the validation cohort. In both the discovery and validation cohort, size and concentration of EV were analyzed by nanoparticle analysis after each EV purification method. However, the samples isolated by PEG precipitation and immunoaffinity were also analyzed by ExoView. This visualization technique depends on immunocapture of EVs to a microarray chip by different EV-enriched surface proteins (74). In the discovery cohort sequencing of RNA and DNA resulted in identification of 4 repetitive elements upregulated in serum from patients with osteosarcoma, in comparison to healthy controls. This finding was then confirmed in the validation cohort. The complex subgrouping and different techniques within the validation weakens the possibility to draw any conclusions. It demands further validation in a patient cohort analyzed with a uniform approach to EV isolation, visualization and characterization. These limitations result in a PedEV score of 55% and EV-METRIC score of 14%. Patient inclusion and exclusion is not clearly described, which precludes assessment of selection bias. The presence of a validation cohort is good, however it is not fully independent to the discovery cohort since 2 samples from the discovery cohort were also analyzed in the validation cohort. Furthermore, the validation cohort is divided in several subgroups with different techniques. This results in a GRADE score of 7.

In summary, several miRNAs were identified in several osteosarcoma studies, including miR-25-3p (82, 83) and miR-21-5p (85, 86). Interestingly, miR-675 (72), miR-148a (69, 85, 86) were found in both *in vivo* and *in vitro* studies. With respect to EV methodology, we found differences in the extent of details reported for EV characterization between the *in vitro* and *in vivo* experiments.

#### Rhabdomyosarcoma

Rhabdomyosarcoma is a highly malignant cancer that develops from skeletal myoblast-like cells (116). Rhabdomyosarcoma is the most common soft tissue sarcoma in children and has a slight male predominance (117). The primary tumor can arise in a variety of anatomical sites, including the head, neck, and extremities, and metastases in the lungs, bone, and/or bone marrow are quite common (118, 119). Two major histological subtypes of rhabdomyosarcoma—embryonal and alveolar—have been identified. Alveolar tumors are often associated with the recurrent chromosomal translocations t(2;13) and t(1;13), which generate fusion oncoproteins between *PAX3* and *FOXO1* and between *PAX7* and *FOXO1*, respectively (120). Although the 5-year overall survival rate is now as high as 70% due to therapeutic advances, the cure rate among patients with metastatic and/or recurrent rhabdomyosarcoma is still low (121). Our literature study identified one study that examined EVs in rhabdomyosarcoma using both *in vivo* and *in vitro* experiments and two additional *in vitro* studies; all three studies were performed by the same group.

In their first study, Ghayad et al. characterized the miRNA expression profiles of EVs secreted by five rhabdomyosarcoma cell lines (**Table 3**) (87). They found miRNAs that were differentially expressed between rhabdomyosarcoma-derived EVs and the corresponding cell lysates, and they also found differential expression between cell lines. Two miRNAs—miR-1246 and miR-1268—were enriched in the EVs of all five rhabdomyosarcoma cell lines. Rhabdomyosarcoma-derived EVs were also shown to increase the proliferation of recipient fibroblasts and rhabdomyosarcoma cells. Moreover, these EVs also induced the migration and invasion of normal fibroblasts, and they promoted angiogenesis in endothelial cells. Subsequently, Rammal et al. examined the protein composition of EVs derived from five rhabdomyosarcoma cell lines using liquid chromatography-MS/MS (LC-MS/MS) (**Table 3**) (88). They found a total of 80 proteins that were common to all five cell lines, as well as 81 that were specific to embryonal rhabdomyosarcoma cells and 42 that were specific to alveolar rhabdomyosarcoma cells. Pathway analysis revealed that these EV proteins are involved in pathways related to tumor cell invasion, proliferation, and metastasis. Thus, these proteins may serve as potential biomarkers, although this should be tested in a clinical study.

Finally, in their recent study, Ghamloush et al. found that expressing the PAX3-FOXO1 fusion protein in murine myoblasts modulated the miRNA content and paracrine function of their EVs, promoting the proliferation, migration, and invasion of recipient fibroblasts (**Tables 2**, **3**) (75). Hierarchical clustering of miRNA microarray profiling data showed that expressing the PAX3-FOXO1 fusion protein altered the EVs’ miRNA content. Interestingly, miR-486-5p was identified as a downstream effector of PAX3-FOXO1 expressed in the EVs of all five rhabdomyosarcoma cell lines, albeit at higher levels in the alveolar rhabdomyosarcoma cell lines compared to the embryonal cell lines. The authors also found this miRNA in serum-derived EVs obtained from patients with rhabdomyosarcoma; in one patient with an alveolar tumor, the levels of miR-486-5p decreased after chemotherapy when the patient was in remission. Despite the relatively small patient cohort, these findings suggest that this miRNA may play a clinically relevant role in patients with rhabdomyosarcoma. A follow-up study with a larger cohort may provide additional insights into the potential use of miR-486-5p as a diagnostic biomarker and for assessing the patient’s response to chemotherapy. However, this study received a GRADE score of only 5, as the patient cohort and inclusion criteria were not described in sufficient detail, and their findings were not validated in an independent cohort.

With respect to the EV methodology for the *in vitro* experiments, these three reports had good EV-METRIC scores (33%, 33%, and 38% for the first, second, and third studies, respectively) and PedEV scores (71.5%, 66%, and 66%, respectively). However, for the *in vivo* experiments EV characterization was not performed, and—importantly—no healthy controls were included.

Overall, miR-486-5p was the only miRNA that was found to be upregulated in the rhabdomyosarcoma-derived EVs isolated from both patient serum samples and cell lines (75). However, given the low number of patients with rhabdomyosarcoma included in this study, additional fundamental work regarding characterization of the EVs is warranted before EV-derived diagnostics can be applied in clinical practice.

#### Ewing Sarcoma

Ewing sarcoma is the second most common bone tumor, mostly presenting in adolescents (122, 123). It is characterized by the presence of a tumor-driving fusion gene, the most common one is EWSR1-FLI1, but several other combinations by members from the FET and ETS gene families have been described, e.g. EWSR1-ERG or FUS-FEV (122). Currently, risk stratification at initial diagnosis relies on imaging and molecular pathology. The first step is often FISH and/or RT-qPCR for the detection of the most common EWSR1 rearrangements (124). Prognosis depends heavily on the presence of metastatic lesions at diagnosis, which mostly presents in the lungs, bone and bone marrow (122). Treatment consists of a combination of chemotherapy, local control by surgery and radiotherapy (122, 123). Evaluation of treatment response is an important challenge, since relapse is associated with <10% 5-years survival (122). Currently, response evaluation depends on imaging. However, liquid biopsies are also gaining attention. The use of cell-free DNA has been explored in several reports (29, 30, 125) but often the level of tumor-derived cell-free DNA is low which limits sensitivity. Detection of circulating tumor cells from blood is also an option, but sensitivity is challenging, due to a high signal-to-noise ratio in peripheral blood cells and not all tumors shedding cells into circulation (34, 124). Considering the limitations of other liquid biopsy-based targets, EVs are also an interesting source of biomarkers in Ewing sarcoma. We identified 3 reports that studied EVs from Ewing sarcoma both *in vivo* and *in vitro*, and 2 that contained only *in vitro* data.

Miller et al. (89) were one of the first in 2013 to demonstrate the presence of the EWSR1 fusion gene in RNA isolated from Ewing sarcoma cell line-derived EV (**Table 3**). They identified several other potential Ewing sarcoma-specific genes through analysis of publicly available array data and then confirmed the presence of this panel in their own EV preparations. They went one step further, using RNAse experiments to show that these mRNA markers are truly present within EV. Lastly, they mixed EVs derived from Ewing sarcoma cell lines with plasma from healthy controls, and were also able to detect these markers. On the contrary, in the plasma from 20 healthy controls without EV, these markers were not present. This study reports the EV methodology in detail, which is reflected by a good PedEV score of 77% and also EV METRIC score is quite good with 25%. No clinical samples were included.

Zhang et al. (90) present a microfluidic, chip-based approach for the quantification of tumor-specific mRNA from EV (**Table 3**). All their experiments were performed on EVs purified from conditioned culture medium originating from Ewing sarcoma cell lines, without any *in vivo* validation. PedEV score was 60.5%, resulting from a detailed reporting on EV-enrichment and characterization, but lacking any report on the analysis of EV-derived protein. EV-METRIC score is 29%, which is quite high and is mostly caused by very detailed reporting on the qualitative and quantitative analysis, and the ultracentrifugation specifics.

Dong et al. (76) present a new technique for purifying EVs from plasma from patients with Ewing sarcoma (**Tables 2**, **3**). In their report, they describe in detail the development, optimization and validation of the ‘ES-EV Click Chip’, first in conditioned culture medium from Ewing sarcoma cell lines. The ES-EV Click Chip combines click chemistry-mediated EV capture within a nanostructure-embedded microchip, which depends on the presence of the protein LINGO1 on Ewing sarcoma-derived EVs. LINGO1 is presented as a Ewing sarcoma-specific marker by the authors. The presence of tumor-specific EVs is then confirmed by RT-ddPCR targeted to the EWSR1 rearrangement. Dong et al. compared this novel ES-EV Click Chip technique to more conventional EV purification approaches, e.g. differential centrifugation, immunocapture and Exoquick. The focus is clearly on the development and optimization of this new technique and the small number of plasma samples included at the end just serves as a small validation. There are no details reported on pre-analytical variables for the plasma samples, such as type of blood tube. Patient characteristics and timing of sampling are also not reported. This results in a low GRADE score of 5. PedEV is more average (55%) since the *in vitro* details are well described, however conventional EV characterization techniques are not reported (or not detailed enough) which leads to an EV-METRIC score of 0%.

Samuel et al. (77) also report on a new approach to isolating Ewing sarcoma-specific EVs (**Tables 2**, **3**). They started by performing proteomics on EVs isolated from different Ewing sarcoma cell lines. By comparing these data to proteomics data from healthy human plasma, they identified Ewing sarcoma-specfic markers CD99 and NGFR. The next step was to develop an immunocapture approach combining CD99 and NGFR and thereby purifying tumor-specific EVs. They confirmed the presence of Ewing sarcoma-specific mRNA by performing RT-qPCR for the EWSR1 fusions. Finally, they performed this Ewing-EV-specific immunocapture on plasma of a small cohort of patients and compared this to healthy controls. It is an impressive effort, however especially the details on the clinical samples (type of blood tube, preparation of plasma) are not reported, as are some details of the Western Blot procedures, resulting in an EV METRIC score of 0% for the *in vivo* and 11% for the *in vitro* part. Within PedEV, *in vivo* and *in vitro* are taken together, which results in a score of 60.5%. Considering the clinical part of the study, patient details are not reported in detail and there is no independent validation cohort, resulting in a GRADE score of 7.

Sun et al. (78) also developed a click chemistry-based approach for the purification of EV (**Tables 2**, **3**). They first optimized this approach in conditioned medium from an Ewing sarcoma cell line, and then validated its *in vivo* potential in plasma from Ewing sarcoma patients and even patients with pancreatic cancer, coupled to a cohort of healthy controls. To confirm that the EVs from patient plasma are originating from the tumor, RT-dPCR is performed for the EWSR1-FLI1 fusion gene. For 2 patients, sequential samples were also tested and the number of EWSR1-FLI1 copies tracks the course of the disease, as is determined by clinical imaging. This is an interesting finding, suggesting a true potential as a minimal residual disease marker for these EVs isolated with click chemistry. Concerning the GRADE score, this report has an average score (8), with one of the most important limitations being a lack of a validation cohort. The reporting of the methodology behind the report is also sound, only characterization of the EV-related proteins is lacking. This is reflected in a PedEV score of 60.5%. However, EV METRIC score for both *in vivo* and *in vitro* experiments is 0%, since the level of details of the EV enrichment and characterization techniques is not sufficient for EV-TRACK.

### Overview of the miRNAs Identified in EVs Derived from Pediatric Solid Tumors, and the Role of the miRNAs in the Hallmarks of Cancer

**Figure 4:**
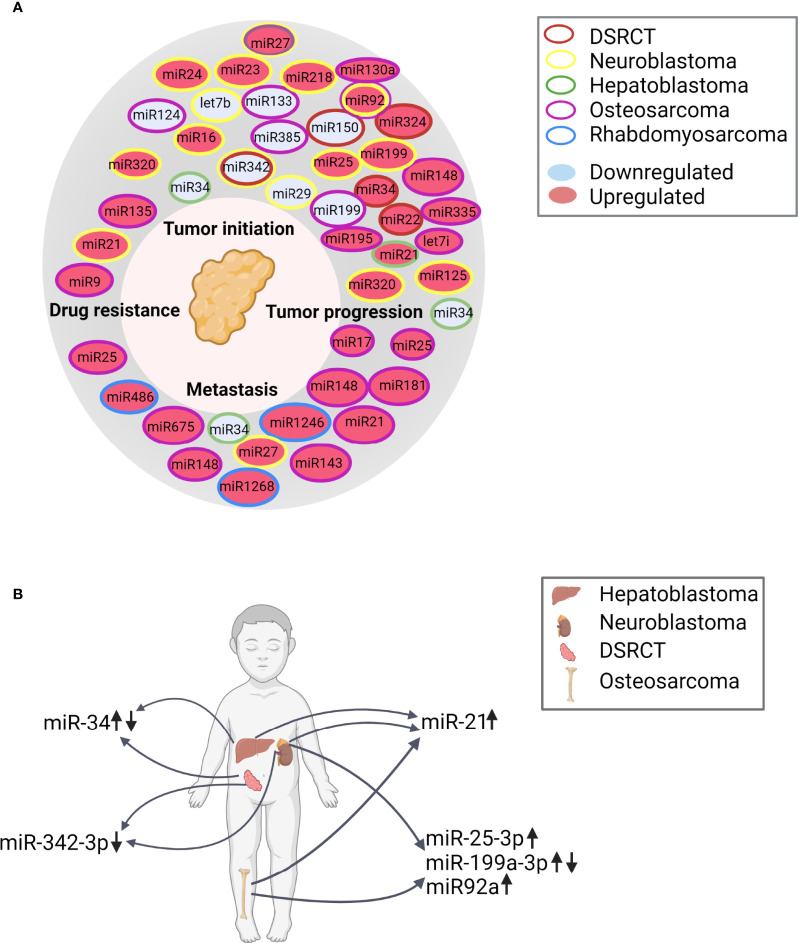
**(A)** Overview of the hallmarks of cancer and the differentially regulated miRNAs described in the various *in vitro* and *in vivo* reports, classified according to their function. DSRCT, desmoplastic small round cell tumor **(B)** Differentially regulated miRNAs in the indicated solid tumors (hepatoblastoma, neuroblastoma, DSRCT, and osteosarcoma) based on the *in vivo* and *in vitro* publications (↑, upregulated; ↓, downregulated). References for miR-21 (65, 79, 80, 85, 86); for miR-25-3p (80, 82, 83); for miR199a-3p (67, 69):; for miR-34 (64, 66); for miR92a (73, 80) and for miR-342-3p (64, 68).

The majority of studies included in our systematic review involved an analysis of miRNA, and nearly all studies reported their putative biological function. This allowed us to provide an overview of the reported miRNAs (both from *in vivo* and *in vitro* studies) in relation to the hallmarks of cancer. In **Figure 4A**, we summarize the miRNAs involved in the “classic” hallmarks of cancer described by Hanahan and Weinberg first in 2000 (126) and again in 2011 (127), and we included an emerging cancer trait: drug resistance (19). In addition, changes in several miRNAs were found in different tumor entities, as illustrated in **Figure 4B**. For example, miR-21—which is known to play a role in metastasis and tumor progression (128)—was upregulated in neuroblastoma (79, 80), hepatoblastoma (65) and osteosarcoma (85, 86). Consistent with this finding, miR-21 has been shown to be overexpressed in many types of solid tumors (129). In addition, miR-25-3p was upregulated in both neuroblastoma (80) and osteosarcoma (82, 83). This miRNA was shown previously to play a role in these two tumor types (130, 131), as well as in other types of cancer, particularly with respect to tumor initiation and progression (132); miR-25-3p has also been reported as a potential biomarker for breast cancer and hepatocarcinoma (133, 134). miR-34a-5p was upregulated in DSRCT (64), while miR-34 miRNAs were downregulated in hepatoblastoma (66). The miR-34 family members play an important role in tumor suppression and are dysregulated in several cancers (135–137). miR-199a-3p was upregulated in neuroblastoma (67) but downregulated in osteosarcoma (69); this miRNA is known to exert opposite effects in different tumors (138), acting as a promoter of leukemic transformation (139) and as a tumor-suppressor gene in both renal cancer (140) and esophageal cancer (141). Finally, miR-342-3p was downregulated in both neuroblastoma (68) and DSRCT (64); this miRNA has been shown to suppress cell proliferation and migration in several types of cancer (142–144).

## Summary and Future Directions

EVs have high potential as diagnostic and prognostic biomarkers for both adult and pediatric cancers (145, 146). However, major discrepancies exist between the number of novel EV-based biomarkers that are reported and the biomarkers that have been successfully incorporated into daily clinical practice, and many obstacles must still be overcome along the road to developing and implementing these biomarkers (147).

Peripheral blood is a suitable source of EVs, as it can be obtained by minimally invasive sampling methods and contains high levels of tumor-derived EVs (148, 149). However, challenges have arisen with respect to the isolation, purification, and analysis of blood-derived EVs. For example, pre-analytical factors such as the type of collection tubes and the conditions used to store the samples can affect several EV characteristics, ranging from the final EV concentration to the origin of the EVs (e.g., platelet-derived versus tumor-derived) (150–154). The method used to enrich EVs from the blood can also affect the subsequent RNA (44, 155, 156) and protein (157, 158) analyses, thereby affecting the final result. Moreover, the complex composition of blood—including non-EV−bound proteins and lipoprotein particles—can complicate the identification of bona fide EV-derived molecules and can potentially hinder the discovery and validation of these biomarkers (159–162). This issue is illustrated by two recent reports by Palviainen et al. (154) and Chiam et al. (163). In their study, Palviainen et al. found that serum contains more platelet-derived EVs compared to plasma; moreover, they found that the protein composition differs between plasma and serum, as well as between samples obtained using different anticoagulants (154). Chiam et al. examined miRNAs in EVs purified from serum and plasma samples obtained from patients with esophageal carcinoma and found that although the plasma contained more miRNA than serum, the plasma also contained more non-EV−derived miRNA (163). With respect to pediatric solid tumors, the clinical studies that we identified from our literature search evaluated EVs that were derived from either serum or plasma; however, detailed descriptions of the pre-analytical factors and the starting sample volumes were often absent, for example in studies involving hepatoblastoma (65, 66) and osteosarcoma (69, 71, 72). Moreover, a wide range of methods were used for enriching and characterizing the EVs, in some cases even within the same publication (67, 70). These missing details limit the studies’ reproducibility and our ability to correctly interpret the resulting data, thereby preventing subsequent validation in a clinical setting.

Our search of the literature for *in vitro* studies assessing EV-derived biomarkers in pediatric solid tumors yielded >3000 hits. However, when focusing on clinical studies that described EVs derived from liquid biopsies from children with solid tumors, and when we evaluated whether these *in vivo* findings were supported using *in vitro* data, we found only the 27 reports that we discussed in this review. It is interesting that we did not find many reports studying the use of microfluidics or nanostructure-based approaches, apart from the two reports in Ewing sarcoma (76, 90), even though in theory these approaches would be suited for low input samples and point-of-care use. Also, more novel particle characterization platforms like Raman scattering (164, 165) were not used in the reports that we found. However, these techniques are often still in early development phases, and pre-clinical testing, which is challenging considering the limited sample number and volumes available in pediatric oncology.

The majority of studies included in this review, were *in vitro* and focused on EVs secreted from cultured cancer cell lines, whereas validation of these biomarkers in physiologically relevant biofluids was often not performed. With respect to the *in vivo* studies, important details regarding the enrichment and characterization platforms of EVs were often not reported, as reflected by the relatively low PedEV scores for these studies. Moreover, many studies did not report using—and therefore may not have used—a density gradient for EV enrichment and/or purification, and they did not report in details on EV characterization, thus resulting in low EV-METRIC scores. Overall, many studies yielded relatively higher scores from PedEV than from EV-TRACK. This is probably caused by the rigorous EV-TRACK scoring system, with points allocated for reporting on specific techniques, e.g. density gradient and details on both qualitative and quantitative analysis. As mentioned before, pediatric studies on patient samples are limited by sample volumes which results in a limitation in the number of techniques that can be performed. The PedEV score requires no specific techniques to be performed and allocates scores for more generally defined criteria (e.g. at least one method for particle characterization not further specified). This also increases the PedEV scores for studies using less conventional EV enrichment approaches, e.g. click chemistry-based approaches. Furthermore, PedEV allocates a general score for the entire report, creating the possibility for a report with less detailed reporting on *in vivo* experiments but with a very detailed report of *in vitro* experiments to still receive a good score. In this respect, it is important to emphasize that EV-TRACK was developed as a general tool for scoring the reproducibility and reporting of EV research and is based on studies using conditioned culture medium or biofluids collected from adults. Given that pediatric studies are far more limited with respect to patient numbers and the volume of biofluids, the extent of EV characterization is limited, as is the inclusion of healthy controls, particularly age-matched controls. Another consideration is that because the field of EV research in pediatric oncology is relatively new and often limited to pediatric oncology centers, EV-specific knowledge and equipment are not yet widely available. Thus, our PedEV score may provide a more lenient and flexible scoring system for EV characterization, at least until the pediatric research community reaches the level of standards that are only now emerging in adult studies involving EVs. Indeed, the EV field is not the first to experience a gap in the quality of study designs between pediatric and adult research (166). Closing this gap will require collaboration beyond the borders of the respective centers and countries, as well as collaboration between scientists in the fields of pediatrics and adult medicine.

Altered regulation of miRNAs has been associated with the initiation and progression of cancer (167). Moreover, the potential of miRNAs was previously demonstrated in adults, with several ongoing clinical trials investigating the potential of using EV-derived miRNAs as diagnostic, predictive, and/or prognostic biomarkers (168). In the studies we evaluated in this review, the same miRNAs were upregulated both *in vivo* and *in vitro* in neuroblastoma (67), osteosarcoma (72), and rhabdomyosarcoma (69, 72, 85, 86). This finding suggests that *in vitro* screening of candidate biomarkers can be highly valuable before moving to *in vivo* validation. However, it is important to note that most of these biomarkers were identified within the same study and/or by the same group. In addition, a study using alveolar rhabdomyosarcoma cell lines suggests that gene expression can differ between *in vitro* conditions and the primary tumor (169). This finding calls into question the value of *in vitro* validation studies, as they may not fully recapitulate the clinical situation. Nevertheless, if *in vitro* studies are performed, we recommend using the same techniques that were used in the corresponding clinical studies, thus reducing technical variations and improving the resulting conclusions. An even better strategy would be to validate the *in vivo* findings in an independent cohort, thus strengthening the claim of identifying a promising new biomarker.

The finding that the same miRNAs are differentially regulated in different tumor types suggests that a panel of miRNAs may be more suitable than any given miRNA as a general pediatric oncology marker, as it may span the entire spectrum of pediatric solid tumors. Studying the changes in this miRNA panel throughout the course of the disease may even lead to the use of miRNAs as a marker of minimal residual disease, as shown previously in adults with Hodgkin lymphoma (4).

To conclude, EVs remain a promising diagnostic biomarker for use in pediatric solid tumors. However, for many tumor types the methodical research—and in particular, *in vivo* validation—is currently lacking. Thus, studies using standardized methods and clear reporting of each step in the enrichment and analysis of EVs derived from liquid biopsies are urgently needed in the field of pediatric oncology. Such studies will likely accelerate both the validation of EV-based techniques and the translation of these biomarkers from the bench to the bedside.

## Data Availability Statement

The original contributions presented in the study are included in the article/**Supplementary Material**. Further inquiries can be directed to the corresponding author.

## Author Contributions

NL, EK, AE-M, EL-A, CO, MW and GT: conceptualization. NL and EK: literature search and writing draft. AE-M, EL-A, CO, MW and GT: review and editing manuscript. All authors contributed to the article and approved the submitted version.

## Funding

NL was supported by Children Cancer Free (KiKa) grant number 312. EL-A is supported by the European Union’s Horizon 2020 research and innovation programme under the Marie Skłodowska-Curie grant agreement No 722148. AE-M, CO, and MW were funded the Perspectief Program Cancer ID [14193], which was in part financed by the Netherlands Organization for Scientific Research–Domain Applied and Engineering Sciences (NWO-TTW).

## Conflict of Interest

The authors declare that the research was conducted in the absence of any commercial or financial relationships that could be construed as a potential conflict of interest.

## Publisher’s Note

All claims expressed in this article are solely those of the authors and do not necessarily represent those of their affiliated organizations, or those of the publisher, the editors and the reviewers. Any product that may be evaluated in this article, or claim that may be made by its manufacturer, is not guaranteed or endorsed by the publisher.

## Abbreviations

EVs, extracellular vesicles; GRADE, Grading of Recommendations, Assessment, Development, and Evaluations; ISEV, International Society for Extracellular Vesicles; KEGG, Kyoto Encyclopedia of Genes and Genomes; MISEV, Minimal Information for Studies of Extracellular Vesicles; MS, mass spectrometry; PedEV, pediatric EV score; RT-ddPCR, reverse transcriptase droplet digital PCR; RT-dPCR, reverse transcriptase digital PCR; RT-qPCR, reverse transcriptase quantitative PCR.

## References

[1] van Niel G D’Angelo G Raposo G . Shedding Light on the Cell Biology of Extracellular Vesicles. Nat Rev Mol Cell Biol (2018) 19(4):213–28. doi: 10.1038/nrm.2017.125 29339798

[2] Bebelman MP Smit MJ Pegtel DM Baglio SR . Biogenesis and Function of Extracellular Vesicles in Cancer. Pharmacol Ther (2018) 188:1–11. doi: 10.1016/j.pharmthera.2018.02.013 29476772

[3] Becker A Thakur BK Weiss JM Kim HS Peinado H Lyden D . Extracellular Vesicles in Cancer: Cell-To-Cell Mediators of Metastasis. Cancer Cell (2016) 30(6):836–48. doi: 10.1016/j.ccell.2016.10.009 PMC5157696 27960084

[4] van Eijndhoven MA Zijlstra JM Groenewegen NJ Drees EE van Niele S Baglio SR . Plasma Vesicle miRNAs for Therapy Response Monitoring in Hodgkin Lymphoma Patients. JCI Insight (2016) 1(19):e89631. doi: 10.1172/jci.insight.89631 27882350 PMC5111516

[5] Merchant ML Rood IM Deegens JKJ Klein JB . Isolation and Characterization of Urinary Extracellular Vesicles: Implications for Biomarker Discovery. Nat Rev Nephrol (2017) 13(12):731–49. doi: 10.1038/nrneph.2017.148 PMC5941934 29081510

[6] Raposo G Stoorvogel W . Extracellular Vesicles: Exosomes, Microvesicles, and Friends. J Cell Biol (2013) 200(4):373–83. doi: 10.1083/jcb.201211138 PMC3575529 23420871

[7] Willms E Cabañas C Mäger I Wood MJA Vader P . Extracellular Vesicle Heterogeneity: Subpopulations, Isolation Techniques, and Diverse Functions in Cancer Progression. Front Immunol (2018) 9:738. doi: 10.3389/fimmu.2018.00738 PMC5936763 29760691

[8] Yuana Y Sturk A Nieuwland R . Extracellular Vesicles in Physiological and Pathological Conditions. Blood Rev (2013) 27(1):31–9. doi: 10.1016/j.blre.2012.12.002 23261067

[9] Zaborowski MP Balaj L Breakefield XO Lai CP . Extracellular Vesicles: Composition, Biological Relevance, and Methods of Study. BioScience (2015) 65(8):783–97. doi: 10.1093/biosci/biv084 PMC4776721 26955082

[10] Simeone P Bologna G Lanuti P Pierdomenico L Guagnano MT Pieragostino D . Extracellular Vesicles as Signaling Mediators and Disease Biomarkers Across Biological Barriers. Int J Mol Sci (2020) 21(7):2514. doi: 10.3390/ijms21072514 PMC7178048 32260425

[11] Caby MP Lankar D Vincendeau-Scherrer C Raposo G Bonnerot C . Exosomal-Like Vesicles are Present in Human Blood Plasma. Int Immunol (2005) 17(7):879–87. doi: 10.1093/intimm/dxh267 15908444

[12] Berckmans RJ Lacroix R Hau CM Sturk A Nieuwland R . Extracellular Vesicles and Coagulation in Blood From Healthy Humans Revisited. J Extracell Vesicles (2019) 8(1):1688936. doi: 10.1080/20013078.2019.1688936 31762964 PMC6853244

[13] Akers J Ramakrishnan V Kim R Phillips S Kaimal V Mao Y . miRNA Contents of Cerebrospinal Fluid Extracellular Vesicles in Glioblastoma Patients. J Neuro-Oncol (2015) 123:205–216. doi: 10.1007/s11060-015-1784-3 PMC4459648 25903655

[14] Gonzales PA Zhou H Pisitkun T Wang NS Star RA Knepper MA . Isolation and Purification of Exosomes in Urine. Methods Mol Biol (2010) 641:89–99. doi: 10.1007/978-1-60761-711-2_6 20407943

[15] Zonneveld MI Brisson AR van Herwijnen MJC Tan S van de Lest CHA Redegeld FA . Recovery of Extracellular Vesicles From Human Breast Milk is Influenced by Sample Collection and Vesicle Isolation Procedures. J Extracell Vesicles (2014) 3. doi: 10.3402/jev.v3.24215 PMC4139932 25206958

[16] Weiser DA West-Szymanski DC Fraint E Weiner S Rivas MA Zhao CWT . Progress Toward Liquid Biopsies in Pediatric Solid Tumors. Cancer Metastasis Rev (2019) 38(4):553–71. doi: 10.1007/s10555-019-09825-1 PMC6995761 31836951

[17] Nakata R Shimada H Fernandez GE Fanter R Fabbri M Malvar J . Contribution of Neuroblastoma-Derived Exosomes to the Production of Pro-Tumorigenic Signals by Bone Marrow Mesenchymal Stromal Cells. J Extracell Vesicles (2017) 6(1):1332941. doi: 10.1080/20013078.2017.1332941 28717423 PMC5505006

[18] Tamura T Yoshioka Y Sakamoto S Ichikawa T Ochiya T . Extracellular Vesicles in Bone Metastasis: Key Players in the Tumor Microenvironment and Promising Therapeutic Targets. Int J Mol Sci (2020) 21(18):6880. doi: 10.3390/ijms21186680 PMC7555648 32932657

[19] Xavier CP Caires HR Barbosa MA Bergantim R Guimarães JE Vasconcelos MH . The Role of Extracellular Vesicles in the Hallmarks of Cancer and Drug Resistance. Cells (2020) 9(5):1141. doi: 10.3390/cells9051141 PMC7290603 32384712

[20] Namee NM O’Driscoll L . Extracellular Vesicles and Anti-Cancer Drug Resistance. Biochim Biophys Acta Rev Cancer (2018) 1870(2):123–36. doi: 10.1016/j.bbcan.2018.07.003 30003999

[21] Nanou A Coumans FAW van Dalum G Zeune LL Dolling D Onstenk W . Circulating Tumor Cells, Tumor-Derived Extracellular Vesicles and Plasma Cytokeratins in Castration-Resistant Prostate Cancer Patients. Oncotarget (2018) 9(27):19283–93. doi: 10.18632/oncotarget.25019 PMC5922396 29721202

[22] Nanou A Miller MC Zeune LL de Wit S Punt CJA Groen HJM . Tumour-Derived Extracellular Vesicles in Blood of Metastatic Cancer Patients Associate With Overall Survival. Br J Cancer (2020) 122(6):801–11. doi: 10.1038/s41416-019-0726-9 PMC7078322 31937922

[23] Kosaka N Kogure A Yamamoto T Urabe F Usuba W Prieto-Vila M . Exploiting the Message From Cancer: The Diagnostic Value of Extracellular Vesicles for Clinical Applications. Exp Mol Med (2019) 51(3):1–9. doi: 10.1038/s12276-019-0219-1 PMC6418231 30872565

[24] Van Paemel R Vlug R De Preter K Van Roy N Speleman F Willems L . The Pitfalls and Promise of Liquid Biopsies for Diagnosing and Treating Solid Tumors in Children: A Review. Eur J Pediatr (2020) 179(2):191–202. doi: 10.1007/s00431-019-03545-y 31897843 PMC6971142

[25] van Zogchel LMJ Lak NSM Verhagen OJHM Tissoudali A Gusmalla Nuru M Gelineau NU . Novel Circulating Hypermethylated RASSF1A ddPCR for Liquid Biopsies in Patients With Pediatric Solid Tumors. JCO Precis Oncol (2021) 5:1738–48. doi: 10.1200/PO.21.00130 PMC8608265 34820594

[26] van Zogchel LMJ van Wezel EM van Wijk J Stutterheim J Bruins WSC Zappeij-Kannegieter L . Hypermethylated RASSF1A as Circulating Tumor DNA Marker for Disease Monitoring in Neuroblastoma. J Clin Oncol Precis Oncol (2020) 4:PO.19.00261. doi: 10.1200/PO.19.00261 PMC7446415 32923888

[27] Lodrini M Graef J Thole-Kliesch TM Astrahantseff K Sprussel A Grimaldi M . Targeted Analysis of Cell-Free Circulating Tumor DNA is Suitable for Early Relapse and Actionable Target Detection in Patients With Neuroblastoma. Clin Cancer Res (2022) 28(9):1809–20. doi: 10.1158/1078-0432.CCR-21-3716 35247920

[28] Lodrini M Sprussel A Astrahantseff K Tiburtius D Konschak R Lode HN . Using Droplet Digital PCR to Analyze MYCN and ALK Copy Number in Plasma From Patients With Neuroblastoma. Oncotarget (2017) 8(49):85234–51. doi: 10.18632/oncotarget.19076 PMC5689606 29156716

[29] Van Paemel R Vandeputte C Raman L Van Thorre J Willems L Van Dorpe J . The Feasibility of Using Liquid Biopsies as a Complementary Assay for Copy Number Aberration Profiling in Routinely Collected Paediatric Cancer Patient Samples. Eur J Cancer (2022) 160:12–23. doi: 10.1016/j.ejca.2021.09.022 34794856

[30] Klega K Imamovic-Tuco A Ha G Clapp AN Meyer S Ward A . Detection of Somatic Structural Variants Enables Quantification and Characterization of Circulating Tumor DNA in Children With Solid Tumors. JCO Precis Oncol (2018) 2018:PO.17.00285. doi: 10.1200/PO.17.00285 PMC6049092 30027144

[31] Lak NSM Voormanns TL Zappeij-Kannegieter L van Zogchel LMJ Fiocco M van Noesel MM . Improving Risk Stratification for Pediatric Patients With Rhabdomyosarcoma by Molecular Detection of Disseminated Disease. Clin Cancer Res (2021) 27(20):5576–85. doi: 10.1158/1078-0432.CCR-21-1083 PMC9401561 34285060

[32] Stutterheim J Gerritsen A Zappeij-Kannegieter L Kleijn I Dee R Hooft L . PHOX2B Is a Novel and Specific Marker for Minimal Residual Disease Testing in Neuroblastoma. J Clin Oncol (2008) 26(33):5443–9. doi: 10.1200/JCO.2007.13.6531 18838715

[33] Stutterheim J Gerritsen A Zappeij-Kannegieter L Yalcin B Dee R van Noesel MM . Detecting Minimal Residual Disease in Neuroblastoma: The Superiority of a Panel of Real-Time Quantitative PCR Markers. Clin Chem (2009) 55(7):1316–26. doi: 10.1373/clinchem.2008.117945 19460840

[34] Tellez-Gabriel M Brown HK Young R Heymann MF Heymann D . The Challenges of Detecting Circulating Tumor Cells in Sarcoma. Front Oncol (2016) 6:202. doi: 10.3389/fonc.2016.00202 27656422 PMC5013264

[35] Chicard M Colmet-Daage L Clement N Danzon A Bohec M Bernard V . Whole-Exome Sequencing of Cell-Free DNA Reveals Temporo-Spatial Heterogeneity and Identifies Treatment-Resistant Clones in Neuroblastoma. Clin Cancer Res (2018) 24(4):939–49. doi: 10.1158/1078-0432.CCR-17-1586 29191970

[36] Eleveld TF Oldridge DA Bernard V Koster J Colmet Daage L Diskin SJ . Relapsed Neuroblastomas Show Frequent RAS-MAPK Pathway Mutations. Nat Genet (2015) 47(8):864–71. doi: 10.1038/ng.3333 PMC4775079 26121087

[37] Van Wezel EM Van Zogchel LMJ Van Wijk J Timmerman I Vo N Zappeij-Kannegieter L . Mesenchymal Neuroblastoma Cells are Undetected by Current mRNA Marker Panels: The Development of a Specific Neuroblastoma Mesenchymal Minimal Residual Disease Panel. J Clin Oncol Precis Oncol (2019) 3:PO.18.00413. doi: 10.1200/PO.18.00413 PMC8133311 34036221

[38] Cui M Wang H Yao X Zhang D Xie Y Cui R . Circulating MicroRNAs in Cancer: Potential and Challenge. Front Genet (2019) 10:626. doi: 10.3389/fgene.2019.00626 31379918 PMC6656856

[39] Mussbacher M Pirabe A Brunnthaler L Schrottmaier WC Assinger A . Horizontal MicroRNA Transfer by Platelets - Evidence and Implications. Front Physiol (2021) 12:678362. doi: 10.3389/fphys.2021.678362 34149456 PMC8209332

[40] Coumans FAW Brisson AR Buzas EI Dignat-George F Drees EEE El-Andaloussi S . Methodological Guidelines to Study Extracellular Vesicles. Circ Res (2017) 120(10):1632–48. doi: 10.1161/CIRCRESAHA.117.309417 28495994

[41] Shirejini SZ Inci F . The Yin and Yang of Exosome Isolation Methods: Conventional Practice, Microfluidics, and Commercial Kits. Biotechnol Adv (2022) 54:107814. doi: 10.1016/j.biotechadv.2021.107814 34389465

[42] Abreu CM Costa-Silva B Reis RL Kundu SC Caballero D . Microfluidic Platforms for Extracellular Vesicle Isolation, Analysis and Therapy in Cancer. Lab Chip (2022) 22(6):1093–125. doi: 10.1039/D2LC00006G 35253032

[43] Singh PK Patel A Kaffenes A Hord C Kesterson D Prakash S . Microfluidic Approaches and Methods Enabling Extracellular Vesicle Isolation for Cancer Diagnostics. Micromachines (Basel) (2022) 13(1):139. doi: 10.3390/mi13010139 35056304 PMC8778688

[44] Van Deun J Mestdagh P Sormunen R Cocquyt V Vermaelen K Vandesompele J . The Impact of Disparate Isolation Methods for Extracellular Vesicles on Downstream RNA Profiling. J Extracell Vesicles (2014) 3. doi: 10.3402/jev.v3.24858 PMC4169610 25317274

[45] Vergauwen G Dhondt B Van Deun J De Smedt E Berx G Timmerman E . Confounding Factors of Ultrafiltration and Protein Analysis in Extracellular Vesicle Research. Sci Rep (2017) 7(1):2704. doi: 10.1038/s41598-017-02599-y 28577337 PMC5457435

[46] Arkesteijn GJ Lozano-Andrés E Libregts SF Wauben MHM . Improved Flow Cytometric Light Scatter Detection of Submicron-Sized Particles by Reduction of Optical Backgrouns Signals. Cytometry A (2020) 97(6):610–9. doi: 10.1002/cyto.a.24036 PMC7384008 32459071

[47] Welsh JA van der Pol E Arkesteijn GJA Bremer M Brisson A Coumans F . MIFlowCyt-EV: A Framework for Standardized Reporting of Extracellular Vesicle Flow Cytometry Experiments. J Extracell Vesicles (2020) 9(1):1713526. doi: 10.1080/20013078.2020.1713526 32128070 PMC7034442

[48] Lotvall J Hill AF Hochberg F Buzas EI Di Vizio D Gardiner C . Minimal Experimental Requirements for Definition of Extracellular Vesicles and Their Functions: A Position Statement From the International Society for Extracellular Vesicles. J Extracell Vesicles (2014) 3:26913. doi: 10.3402/jev.v3.26913 25536934 PMC4275645

[49] Théry C Witwer KW Aikawa E Alcaraz MJ Anderson JD Andriantsitohaina R . Minimal Information for Studies of Extracellular Vesicles 2018 (MISEV2018): A Position Statement of the International Society for Extracellular Vesicles and Update of the MISEV2014 Guidelines. J Extracell Vesicles (2018) 7(1):1535750. doi: 10.1080/20013078.2018.1535750 30637094 PMC6322352

[50] Consortium E-T Van Deun J Mestdagh P Agostinis P Akay O Anand S . EV-TRACK: Transparent Reporting and Centralizing Knowledge in Extracellular Vesicle Research. Nat Methods (2017) 14(3):228–32. doi: 10.1038/nmeth.4185 28245209

[51] Van Deun J Hendrix A Consortium E-T . Is Your Article EV-TRACKed? J Extracell Vesicles (2017) 6(1):1379835. doi: 10.1080/20013078.2017.1379835 29184624 PMC5698936

[52] Peng J Wang W Hua S Liu L . Roles of Extracellular Vesicles in Metastatic Breast Cancer. Breast Cancer (Auckl) (2018) 12:1178223418767666. doi: 10.1177/1178223418767666 29881285 PMC5987895

[53] Linxweiler J Junker K . Extracellular Vesicles in Urological Malignancies: An Update. Nat Rev Urol (2020) 17(1):11–27. doi: 10.1038/s41585-019-0261-8 31827264

[54] Tang MK Wong AS . Exosomes: Emerging Biomarkers and Targets for Ovarian Cancer. Cancer Lett (2015) 367(1):26–33. doi: 10.1016/j.canlet.2015.07.014 26189430

[55] Skog J Wurdinger T van Rijn S Meijer DH Gainche L Sena-Esteves M . Glioblastoma Microvesicles Transport RNA and Proteins That Promote Tumour Growth and Provide Diagnostic Biomarkers. Nat Cell Biol (2008) 10(12):1470–6. doi: 10.1038/ncb1800 PMC3423894 19011622

[56] Kadota T Yoshioka Y Fujita Y Kuwano K Ochiya T . Extracellular Vesicles in Lung Cancer—From Bench to Bedside. Semin Cell Dev Biol (2017) 67:39–47. doi: 10.1016/j.semcdb.2017.03.001 28267596

[57] Lee RS Stewart C Carter SL Ambrogio L Cibulskis K Sougnez C . A Remarkably Simple Genome Underlies Highly Malignant Pediatric Rhabdoid Cancers. J Clin Invest (2012) 122(8):2983–8. doi: 10.1172/JCI64400 PMC3408754 22797305

[58] Crompton BD Stewart C Taylor-Weiner A Alexe G Kurek KC Calicchio ML . The Genomic Landscape of Pediatric Ewing Sarcoma. Cancer Discov (2014) 4(11):1326–41. doi: 10.1158/2159-8290.CD-13-1037 25186949

[59] Pugh TJ Morozova O Attiyeh EF Asgharzadeh S Wei JS Auclair D . The Genetic Landscape of High-Risk Neuroblastoma. Nat Genet (2013) 45(3):279–84. doi: 10.1038/ng.2529 PMC3682833 23334666

[60] Ma X Liu Y Liu Y Alexandrov LB Edmonson MN Gawad C . Pan-Cancer Genome and Transcriptome Analyses of 1,699 Paediatric Leukaemias and Solid Tumours. Nature (2018) 555(7696):371–6. doi: 10.1038/nature25795 PMC5854542 29489755

[61] Gröbner SN Worst BC Weischenfeldt J Buchhalter I Kleinheinz K Rudneva VA . The Landscape of Genomic Alterations Across Childhood Cancers. Nature (2018) 555(7696):321–7. doi: 10.1038/nature25480 29489754

[62] Guyatt GH Oxman AD Vist GE Kunz R Falck-Ytter Y Alonso-Coello P . GRADE: An Emerging Consensus on Rating Quality of Evidence and Strength of Recommendations. BMJ (2008) 336(7650):924–6. doi: 10.1136/bmj.39489.470347.AD PMC2335261 18436948

[63] GRADE working group . Available at: https://www.gradeworkinggroup.org/.

[64] Colletti M Paolini A Galardi A Di Paolo V Pascucci L Russo I . Expression Profiles of Exosomal miRNAs Isolated From Plasma of Patients With Desmoplastic Small Round Cell Tumor. Epigenomics (2019) 11(5):489–500. doi: 10.2217/epi-2018-0179 30569756

[65] Liu W Chen S Liu B . Diagnostic and Prognostic Values of Serum Exosomal microRNA-21 in Children With Hepatoblastoma: A Chinese Population-Based Study. Pediatr Surg Int (2016) 32(11):1059–65. doi: 10.1007/s00383-016-3960-8 27601233

[66] Jiao C Jiao X Zhu A Ge J Xu X . Exosomal miR-34s Panel as Potential Novel Diagnostic and Prognostic Biomarker in Patients With Hepatoblastoma. J Pediatr Surg (2017) 52(4):618–24. doi: 10.1016/j.jpedsurg.2016.09.070 28277300

[67] Ma J Xu M Yin M Hong J Chen H Gao Y . Exosomal Hsa-Mir199a-3p Promotes Proliferation and Migration in Neuroblastoma. Front Oncol (2019) 9:459. doi: 10.3389/fonc.2019.00459 31249805 PMC6582313

[68] Morini M Cangelosi D Segalerba D Marimpietri D Raggi F Castellano A . Exosomal microRNAs From Longitudinal Liquid Biopsies for the Prediction of Response to Induction Chemotherapy in High-Risk Neuroblastoma Patients: A Proof of Concept SIOPEN Study. Cancers (Basel) (2019) 11(10):1476. doi: 10.3390/cancers11101476 PMC6826693 31575060

[69] Xu JF Wang YP Zhang SJ Chen Y Gu HF Dou XF . Exosomes Containing Differential Expression of microRNA and mRNA in Osteosarcoma That can Predict Response to Chemotherapy. Oncotarget (2017) 8(44):75968–78. doi: 10.18632/oncotarget.18373 PMC5652678 29100284

[70] Baglio SR Lagerweij T Perez-Lanzon M Ho XD Leveille N Melo SA . Blocking Tumor-Educated MSC Paracrine Activity Halts Osteosarcoma Progression. Clin Cancer Res (2017) 23(14):3721–33. doi: 10.1158/1078-0432.CCR-16-2726 28053020

[71] Shen RK Zhu X Yi H Wu CY Chen F Dai LQ . Proteomic Identification of Osteosarcoma-Derived Exosomes and Their Activation of Pentose Phosphate Pathway. Int J Clin Exp Pathol (2016) 9(3):4140–8.

[72] Gong L Bao Q Hu C Wang J Zhou Q Wei L . Exosomal miR-675 From Metastatic Osteosarcoma Promotes Cell Migration and Invasion by Targeting CALN1. Biochem Biophys Res Commun (2018) 500(2):170–6. doi: 10.1016/j.bbrc.2018.04.016 29626470

[73] Ye Z Zheng Z Peng L . MicroRNA Profiling of Serum Exosomes in Patients With Osteosarcoma by High-Throughput Sequencing. J Investig Med (2020) 68(4):893–901. doi: 10.1136/jim-2019-001196 32060049

[74] Cambier L Stachelek K Triska M Jubran R Huang M Li W . Extracellular Vesicle-Associated Repetitive Element DNAs as Candidate Osteosarcoma Biomarkers. Sci Rep (2021) 11(1):94. doi: 10.1038/s41598-020-77398-z 33420117 PMC7794510

[75] Ghamloush F Ghayad SE Rammal G Fahs A Ayoub AJ Merabi Z . The PAX3-FOXO1 Oncogene Alters Exosome miRNA Content and Leads to Paracrine Effects Mediated by Exosomal miR-486. Sci Rep (2019) 9(1):14242. doi: 10.1038/s41598-019-50592-4 31578374 PMC6775163

[76] Dong J Zhang RY Sun N Hu J Smalley MD Zhou A . Coupling Nanostructured Microchips With Covalent Chemistry Enables Purification of Sarcoma-Derived Extracellular Vesicles for Downstream Functional Studies. Adv Funct Mater (2020) 30(49):20033237. doi: 10.1002/adfm.202003237 PMC8248519 34220409

[77] Samuel G Crow J Klein JB Merchant ML Nissen E Koestler DC . Ewing Sarcoma Family of Tumors-Derived Small Extracellular Vesicle Proteomics Identify Potential Clinical Biomarkers. Oncotarget (2020) 11(31):2995–3012. doi: 10.18632/oncotarget.27678 32821345 PMC7415402

[78] Sun N Tran BV Peng Z Wang J Zhang C Yang P . Coupling Lipid Labeling and Click Chemistry Enables Isolation of Extracellular Vesicles for Noninvasive Detection of Oncogenic Gene Alterations. Adv Sci (Weinh) (2022) e2105853. doi: 10.1002/advs.202105853 35486030 PMC9108594

[79] Challagundla KB Wise PM Neviani P Chava H Murtadha M Xu T . Exosome-Mediated Transfer of microRNAs Within the Tumor Microenvironment and Neuroblastoma Resistance to Chemotherapy. J Natl Cancer Inst (2015) 107(7):djv135. doi: 10.1093/jnci/djv135 25972604 PMC4651042

[80] Haug BH Hald OH Utnes P Roth SA Lokke C Flaegstad T . Exosome-Like Extracellular Vesicles From MYCN-Amplified Neuroblastoma Cells Contain Oncogenic miRNAs. Anticancer Res (2015) 35(5):2521–30. 25964525

[81] Jerez S Araya H Thaler R Charlesworth MC López-Solís R Kalergis AM . Proteomic Analysis of Exosomes and Exosome-Free Conditioned Media From Human Osteosarcoma Cell Lines Reveals Secretion of Proteins Related to Tumor Progression. J Cell Biochem (2017) 118(2):351–60. doi: 10.1002/jcb.25642 27356893

[82] Yoshida A Fujiwara T Uotani K Morita T Kiyono M Yokoo S . Clinical and Functional Significance of Intracellular and Extracellular microRNA-25-3p in Osteosarcoma. Acta Med Okayama (2018) 72(2):165–74. doi: 10.18926/AMO/55857 29674765

[83] Fujiwara T Uotani K Yoshida A Morita T Nezu Y Kobayashi E . Clinical Significance of Circulating miR-25-3p as a Novel Diagnostic and Prognostic Biomarker in Osteosarcoma. Oncotarget (2017) 8(20):33375–92. doi: 10.18632/oncotarget.16498 PMC5464875 28380419

[84] Macklin R Wang H Loo D Martin S Cumming A Cai N . Extracellular Vesicles Secreted by Highly Metastatic Clonal Variants of Osteosarcoma Preferentially Localize to the Lungs and Induce Metastatic Behaviour in Poorly Metastatic Clones. Oncotarget (2016) 7(28):43570–87. doi: 10.18632/oncotarget.9781 PMC5190045 27259278

[85] Jerez S Araya H Hevia D Irarrazaval CE Thaler R van Wijnen AJ . Extracellular Vesicles From Osteosarcoma Cell Lines Contain miRNAs Associated With Cell Adhesion and Apoptosis. Gene (2019) 710:246–57. doi: 10.1016/j.gene.2019.06.005 PMC6684290 31176732

[86] Raimondi L De Luca A Gallo A Costa V Russelli G Cuscino N . Osteosarcoma Cell-Derived Exosomes Affect Tumor Microenvironment by Specific Packaging of microRNAs. Carcinogenesis (2020) 41(5):666–77. doi: 10.1093/carcin/bgz130 31294446

[87] Ghayad SE Rammal G Ghamloush F Basma H Nasr R Diab-Assaf M . Exosomes Derived From Embryonal and Alveolar Rhabdomyosarcoma Carry Differential miRNA Cargo and Promote Invasion of Recipient Fibroblasts. Sci Rep (2016) 6:37088. doi: 10.1038/srep37088 27853183 PMC5112573

[88] Rammal G Fahs A Kobeissy F Mechref Y Zhao J Zhu R . Proteomic Profiling of Rhabdomyosarcoma-Derived Exosomes Yield Insights Into Their Functional Role in Paracrine Signaling. J Proteome Res (2019) 18(10):3567–79. doi: 10.1021/acs.jproteome.9b00157 31448612

[89] Miller IV Raposo G Welsch U Prazeres da Costa O Thiel U Lebar M . First Identification of Ewing’s Sarcoma-Derived Extracellular Vesicles and Exploration of Their Biological and Potential Diagnostic Implications. Biol Cell (2013) 105(7):289–303. doi: 10.1111/boc.201200086 23521563

[90] Zhang P Crow J Lella D Zhou X Samuel G Godwin AK . Ultrasensitive Quantification of Tumor mRNAs in Extracellular Vesicles With an Integrated Microfluidic Digital Analysis Chip. Lab Chip (2018) 18(24):3790–801. doi: 10.1039/C8LC01071D PMC6310142 30474100

[91] Thomas R Rajeswaran G Thway K Benson C Shahabuddin K Moskovic E . Desmoplastic Small Round Cell Tumour: The Radiological, Pathological and Clinical Features. Insights Imaging (2013) 4(1):111–8. doi: 10.1007/s13244-012-0212-x PMC3579986 23307783

[92] Gerald WL Ladanyi M de Alava E Cuatrecasas M Kushner BH LaQuaglia MP . Clinical, Pathologic, and Molecular Spectrum of Tumors Associated With T (11; 22)(P13; Q12): Desmoplastic Small Round-Cell Tumor and its Variants. J Clin Oncol (1998) 16(9):3028–36. doi: 10.1200/JCO.1998.16.9.3028 9738572

[93] Gerald WL Haber DA . The EWS-WT1 Gene Fusion in Desmoplastic Small Round Cell Tumor. Semin Cancer Biol (2005) 15(3):197–205. doi: 10.1016/j.semcancer.2005.01.005 15826834

[94] Gedminas JM Chasse MH McBrairty M Beddows I Kitchen-Goosen SM Grohar PJ . Desmoplastic Small Round Cell Tumor is Dependent on the EWS-WT1 Transcription Factor. Oncogenesis (2020) 9(4):41. doi: 10.1038/s41389-020-0224-1 32345977 PMC7188842

[95] Lal DR Su WT Wolden SL Loh KC Modak S La Quaglia MP . Results of Multimodal Treatment for Desmoplastic Small Round Cell Tumors. J Pediatr Surg (2005) 40(1):251–5. doi: 10.1016/j.jpedsurg.2004.09.046 15868593

[96] Bent MA Padilla BE Goldsby RE DuBois SG . Clinical Characteristics and Outcomes of Pediatric Patients With Desmoplastic Small Round Cell Tumor. Rare Tumors (2016) 8(1):6145. doi: 10.4081/rt.2016.6145 27134714 PMC4827651

[97] Aronson DC Meyers RL . Malignant Tumors of the Liver in Children. Semin Pediatr Surg (2016) 25(5):265–75. doi: 10.1053/j.sempedsurg.2016.09.002 27955729

[98] Sumazin P Chen Y Treviño LR Sarabia SF Hampton OA Patel K . Genomic Analysis of Hepatoblastoma Identifies Distinct Molecular and Prognostic Subgroups. Hepatology (2017) 65(1):104–21. doi: 10.1002/hep.28888 27775819

[99] Czauderna P Lopez-Terrada D Hiyama E Häberle B Malogolowkin MH Meyers RL . Hepatoblastoma State of the Art: Pathology, Genetics, Risk Stratification, and Chemotherapy. Curr Opin Pediatr (2014) 26(1):19–28. doi: 10.1097/MOP.0000000000000046 24322718

[100] Horton JD Lee S Brown SR Bader J Meier DE . Survival Trends in Children With Hepatoblastoma. Pediatr Surg Int (2009) 25(5):407. doi: 10.1007/s00383-009-2349-3 19308432

[101] Meyers RL Maibach R Hiyama E Haberle B Krailo M Rangaswami A . Risk-Stratified Staging in Paediatric Hepatoblastoma: A Unified Analysis From the Children’s Hepatic Tumors International Collaboration. Lancet Oncol (2017) 18(1):122–31. doi: 10.1016/S1470-2045(16)30598-8 PMC5650231 27884679

[102] Matthay KK Maris JM Schleiermacher G Nakagawara A Mackall CL Diller L . Neuroblastoma. Nat Rev Dis Primers (2016) 2:16078. doi: 10.1038/nrdp.2016.78 27830764

[103] Louis CU Shohet JM . Neuroblastoma: Molecular Pathogenesis and Therapy. Annu Rev Med (2015) 66:49–63. doi: 10.1146/annurev-med-011514-023121 25386934 PMC4418018

[104] Ahmed AA Zhang L Reddivalla N Hetherington M . Neuroblastoma in Children: Update on Clinicopathologic and Genetic Prognostic Factors. Pediatr Hematol Oncol (2017) 34(3):165–85. doi: 10.1080/08880018.2017.1330375 28662353

[105] Mossé YP Laudenslager M Longo L Cole KA Wood A Attiyeh EF . Identification of ALK as a Major Familial Neuroblastoma Predisposition Gene. Nature (2008) 455(7215):930–5. doi: 10.1038/nature07261 PMC2672043 18724359

[106] Trochet D Bourdeaut F Janoueix-Lerosey I Deville A de Pontual L Schleiermacher G . Germline Mutations of the Paired-Like Homeobox 2B (PHOX2B) Gene in Neuroblastoma. Am J Hum Genet (2004) 74(4):761–4. doi: 10.1086/383253 PMC1181953 15024693

[107] Deyell RJ Attiyeh EF . Advances in the Understanding of Constitutional and Somatic Genomic Alterations in Neuroblastoma. Cancer Genet (2011) 204(3):113–21. doi: 10.1016/j.cancergen.2011.03.001 21504710

[108] Schleiermacher G Janoueix-Lerosey I Ribeiro A Klijanienko J Couturier J Pierron G . Accumulation of Segmental Alterations Determines Progression in Neuroblastoma. J Clin Oncol (2010) 28(19):3122–30. doi: 10.1200/JCO.2009.26.7955 20516441

[109] Mirabello L Troisi RJ Savage SA . Osteosarcoma Incidence and Survival Rates From 1973 to 2004: Data From the Surveillance, Epidemiology, and End Results Program. Cancer: Interdiscip Int J Am Cancer Soc (2009) 115(7):1531–43. doi: 10.1002/cncr.24121 PMC2813207 19197972

[110] Geller DS Gorlick R . Osteosarcoma: A Review of Diagnosis, Management, and Treatment Strategies. Clin Adv Hematol Oncol (2010) 8(10):705–18. 21317869

[111] Luetke A Meyers PA Lewis I Juergens H . Osteosarcoma Treatment–Where do We Stand? A State of the Art Review. Cancer Treat Rev (2014) 40(4):523–32. doi: 10.1016/j.ctrv.2013.11.006 24345772

[112] Klein MJ Siegal GP . Osteosarcoma: Anatomic and Histologic Variants. Am J Clin Pathol (2006) 125(4):555–81. doi: 10.1309/UC6KQHLD9LV2KENN 16627266

[113] Martin JW Squire JA Zielenska M . The Genetics of Osteosarcoma. Sarcoma (2012) 2012:627254–. doi: 10.1155/2012/627254 PMC3364016 22685381

[114] Chen X Bahrami A Pappo A Easton J Dalton J Hedlund E . Recurrent Somatic Structural Variations Contribute to Tumorigenesis in Pediatric Osteosarcoma. Cell Rep (2014) 7(1):104–12. doi: 10.1016/j.celrep.2014.03.003 PMC4096827 24703847

[115] Simpson E Brown HL . Understanding Osteosarcomas. J Am Acad PAs (2018) 31(8):15–9. doi: 10.1097/01.JAA.0000541477.24116.8d 29979330

[116] Skapek SX Ferrari A Gupta AA Lupo PJ Butler E Shipley J . Rhabdomyosarcoma. Nat Rev Dis Primers (2019) 5(1):1. doi: 10.1038/s41572-018-0051-2 30617281 PMC7456566

[117] Ognjanovic S Linabery AM Charbonneau B Ross JA . Trends in Childhood Rhabdomyosarcoma Incidence and Survival in the United States, 1975-2005. Cancer: Interdiscip Int J Am Cancer Soc (2009) 115(18):4218–26. doi: 10.1002/cncr.24465 PMC2953716 19536876

[118] Oberlin O Rey A Lyden E Bisogno G Stevens MC Meyer WH . Prognostic Factors in Metastatic Rhabdomyosarcomas: Results of a Pooled Analysis From United States and European Cooperative Groups. J Clin Oncol (2008) 26(14):2384–9. doi: 10.1200/JCO.2007.14.7207 PMC4558625 18467730

[119] Dasgupta R Fuchs J Rodeberg D . Rhabdomyosarcoma. Semin Pediatr Surg (2016) 25(5):276–83. doi: 10.1053/j.sempedsurg.2016.09.011 27955730

[120] Parham DM Barr FG . Classification of Rhabdomyosarcoma and its Molecular Basis. Adv Anat Pathol (2013) 20(6):387–97. doi: 10.1097/PAP.0b013e3182a92d0d PMC6637949 24113309

[121] Rodeberg D Paidas C . Childhood Rhabdomyosarcoma. Semin Pediatr Surg (2006) 15(1):57–62. doi: 10.1053/j.sempedsurg.2005.11.009 16458847

[122] Grunewald TGP Cidre-Aranaz F Surdez D Tomazou EM de Alava E Kovar H . Ewing Sarcoma. Nat Rev Dis Primers (2018) 4(1):5. doi: 10.1038/s41572-018-0003-x 29977059

[123] Zollner SK Amatruda JF Bauer S Collaud S de Alava E DuBois SG . Ewing Sarcoma-Diagnosis, Treatment, Clinical Challenges and Future Perspectives. J Clin Med (2021) 10(8):1685. doi: 10.3390/jcm10081685 33919988 PMC8071040

[124] Salguero-Aranda C Amaral AT Olmedo-Pelayo J Diaz-Martin J Alava E . Breakthrough Technologies Reshape the Ewing Sarcoma Molecular Landscape. Cells (2020) 9(4):804. doi: 10.3390/cells9040804 PMC7226764 32225029

[125] Van Paemel R De Koker A Vandeputte C van Zogchel L Lammens T Laureys G . Minimally Invasive Classification of Paediatric Solid Tumours Using Reduced Representation Bisulphite Sequencing of Cell-Free DNA: A Proof-of-Principle Study. Epigenetics (2021) 16(2):196–208. doi: 10.1080/15592294.2020.1790950 32662719 PMC7889189

[126] Hanahan D Weinberg RA . The Hallmarks of Cancer. Cell (2000) 100(1):57–70. doi: 10.1016/S0092-8674(00)81683-9 10647931

[127] Hanahan D Weinberg RA . Hallmarks of Cancer: The Next Generation. Cell (2011) 144(5):646–74. doi: 10.1016/j.cell.2011.02.013 21376230

[128] Bautista-Sanchez D Arriaga-Canon C Pedroza-Torres A de la Rosa-Velazquez IA Gonzalez-Barrios R Contreras-Espinosa L . The Promising Role of miR-21 as a Cancer Biomarker and Its Importance in RNA-Based Therapeutics. Mol Ther Nucleic Acids (2020) 20:409–20. doi: 10.1016/j.omtn.2020.03.003 PMC7118281 32244168

[129] Volinia S Calin GA Liu CG Ambs S Cimmino A Petrocca F . A microRNA Expression Signature of Human Solid Tumors Defines Cancer Gene Targets. Proc Natl Acad Sci USA (2006) 103(7):2257–61. doi: 10.1073/pnas.0510565103 PMC1413718 16461460

[130] Wang XH Cai P Wang MH Wang Z . microRNA−25 Promotes Osteosarcoma Cell Proliferation by Targeting the Cell−Cycle Inhibitor P27. Mol Med Rep (2014) 10(2):855–9. doi: 10.3892/mmr.2014.2260 24859599

[131] Ren C Li C Zhang H Gao L Li A . The Role of miR-25 in Pediatric Neuroblastoma. BioMed Res (2017) 28(16):7261–7.

[132] Ding X Zhong T Jiang L Huang J Xia Y Hu R . miR-25 Enhances Cell Migration and Invasion in non-Small-Cell Lung Cancer Cells *via* ERK Signaling Pathway by Inhibiting KLF4. Mol Med Rep (2018) 17(5):7005–16. doi: 10.3892/mmr.2018.8772 PMC5928655 29568911

[133] Hu Z Dong J Wang L-E Ma H Liu J Zhao Y . Serum microRNA Profiling and Breast Cancer Risk: The Use of miR-484/191 as Endogenous Controls. Carcinogenesis (2012) 33(4):828–34. doi: 10.1093/carcin/bgs030 22298638

[134] Li LM Hu ZB Zhou ZX Chen X Liu FY Zhang JF . Serum microRNA Profiles Serve as Novel Biomarkers for HBV Infection and Diagnosis of HBV-Positive Hepatocarcinoma. Cancer Res (2010) 70(23):9798–807. doi: 10.1158/0008-5472.CAN-10-1001 21098710

[135] Guessous F Zhang Y Kofman A Catania A Li Y Schiff D . microRNA-34a is Tumor Suppressive in Brain Tumors and Glioma Stem Cells. Cell Cycle (2010) 9(6):1031–6. doi: 10.4161/cc.9.6.10987 PMC3278213 20190569

[136] Jafari N Abediankenari S . MicroRNA-34 Dysregulation in Gastric Cancer and Gastric Cancer Stem Cell. Tumour Biol (2017) 39(5):1010428317701652. doi: 10.1177/1010428317701652 28468587

[137] Imani S Wu RC Fu J . MicroRNA-34 Family in Breast Cancer: From Research to Therapeutic Potential. J Cancer (2018) 9(20):3765–75. doi: 10.7150/jca.25576 PMC6216011 30405848

[138] Gu S Chan WY . Flexible and Versatile as a Chameleon-Sophisticated Functions of microRNA-199a. Int J Mol Sci (2012) 13(7):8449–66. doi: 10.3390/ijms13078449 PMC3430244 22942713

[139] Alemdehy MF Haanstra JR de Looper HW van Strien PM Verhagen-Oldenampsen J Caljouw Y . ICL-Induced Mir139-3p and Mir199a-3p Have Opposite Roles in Hematopoietic Cell Expansion and Leukemic Transformation. Blood (2015) 125(25):3937–48. doi: 10.1182/blood-2014-11-612507 25778535

[140] Tsukigi M Bilim V Yuuki K Ugolkov A Naito S Nagaoka A . Re-Expression of miR-199a Suppresses Renal Cancer Cell Proliferation and Survival by Targeting GSK-3β. Cancer Lett (2012) 315(2):189–97. doi: 10.1016/j.canlet.2011.10.008 22093618

[141] Phatak P Burrows WM Chesnick IE Tulapurkar ME Rao JN Turner DJ . MiR-199a-3p Decreases Esophageal Cancer Cell Proliferation by Targeting P21 Activated Kinase 4. Oncotarget (2018) 9(47):28391–407. doi: 10.18632/oncotarget.25375 PMC6033339 29983868

[142] Xue X Fei X Hou W Zhang Y Liu L Hu R . miR-342-3p Suppresses Cell Proliferation and Migration by Targeting AGR2 in non-Small Cell Lung Cancer. Cancer Lett (2018) 412:170–8. doi: 10.1016/j.canlet.2017.10.024 29107102

[143] Li XR Chu HJ Lv T Wang L Kong SF Dai SZ . miR-342-3p Suppresses Proliferation, Migration and Invasion by Targeting FOXM1 in Human Cervical Cancer. FEBS Lett (2014) 588(17):3298–307. doi: 10.1016/j.febslet.2014.07.020 25066298

[144] Zhao L Zhang Y . miR-342-3p Affects Hepatocellular Carcinoma Cell Proliferation *via* Regulating NF-κb Pathway. Biochem Biophys Res Commun (2015) 457(3):370–7. doi: 10.1016/j.bbrc.2014.12.119 25580008

[145] Galardi A Colletti M Di Paolo V Vitullo P Antonetti L Russo I . Exosomal MiRNAs in Pediatric Cancers. Int J Mol Sci (2019) 20(18):4600. doi: 10.3390/ijms20184600 PMC6770697 31533332

[146] Xu R Rai A Chen M Suwakulsiri W Greening DW Simpson RJ . Extracellular Vesicles in Cancer—Implications for Future Improvements in Cancer Care. Nat Rev Clin Oncol (2018) 15(10):617. doi: 10.1038/s41571-018-0036-9 29795272

[147] Yekula A Muralidharan K Kang KM Wang L Balaj L Carter BS . From Laboratory to Clinic: Translation of Extracellular Vesicle Based Cancer Biomarkers. Methods (2020) 177:58–66. doi: 10.1016/j.ymeth.2020.02.003 32061674 PMC7198349

[148] Logozzi M De Milito A Lugini L Borghi M Calabrò L Spada M . High Levels of Exosomes Expressing CD63 and Caveolin-1 in Plasma of Melanoma Patients. PloS One (2009) 4(4):e5219. doi: 10.1371/journal.pone.0005219 19381331 PMC2667632

[149] Eldh M Olofsson Bagge R Lässer C Svanvik J Sjöstrand M Mattsson J . MicroRNA in Exosomes Isolated Directly From the Liver Circulation in Patients With Metastatic Uveal Melanoma. BMC Cancer (2014) 14:962. doi: 10.1186/1471-2407-14-962 25510783 PMC4320618

[150] Momen-Heravi F Balaj L Alian S Trachtenberg AJ Hochberg FH Skog J . Impact of Biofluid Viscosity on Size and Sedimentation Efficiency of the Isolated Microvesicles. Front Physiol (2012) 3:162. doi: 10.3389/fphys.2012.00162 22661955 PMC3362089

[151] Menck K Bleckmann A Wachter A Hennies B Ries L Schulz M . Characterisation of Tumour-Derived Microvesicles in Cancer Patients’ Blood and Correlation With Clinical Outcome. J Extracell Vesicles (2017) 6(1):1340745. doi: 10.1080/20013078.2017.1340745 28804596 PMC5533131

[152] Ramirez MI Amorim MG Gadelha C Milic I Welsh JA Freitas VM . Technical Challenges of Working With Extracellular Vesicles. Nanoscale (2018) 10(3):881–906. doi: 10.1039/C7NR08360B 29265147

[153] Clayton A Boilard E Buzas EI Cheng L Falcon-Perez JM Gardiner C . Considerations Towards a Roadmap for Collection, Handling and Storage of Blood Extracellular Vesicles. J Extracell Vesicles (2019) 8(1):1647027. doi: 10.1080/20013078.2019.1647027 31489143 PMC6711123

[154] Palviainen M Saraswat M Varga Z Kitka D Neuvonen M Puhka M . Extracellular Vesicles From Human Plasma and Serum are Carriers of Extravesicular Cargo-Implications for Biomarker Discovery. PloS One (2020) 15(8):e0236439. doi: 10.1371/journal.pone.0236439 32813744 PMC7446890

[155] Helwa I Cai J Drewry MD Zimmerman A Dinkins MB Khaled ML . A Comparative Study of Serum Exosome Isolation Using Differential Ultracentrifugation and Three Commercial Reagents. PloS One (2017) 12(1):e0170628. doi: 10.1371/journal.pone.0170628 28114422 PMC5256994

[156] Andreu Z Rivas E Sanguino-Pascual A Lamana A Marazuela M González-Alvaro I . Comparative Analysis of EV Isolation Procedures for miRNAs Detection in Serum Samples. J Extracell Vesicles (2016) 5(1):31655. doi: 10.3402/jev.v5.31655 27330048 PMC4916259

[157] Kalra H Adda CG Liem M Ang CS Mechler A Simpson RJ . Comparative Proteomics Evaluation of Plasma Exosome Isolation Techniques and Assessment of the Stability of Exosomes in Normal Human Blood Plasma. Proteomics (2013) 13(22):3354–64. doi: 10.1002/pmic.201300282 24115447

[158] Macías M Rebmann V Mateos B Varo N Perez-Gracia JL Alegre E . Comparison of Six Commercial Serum Exosome Isolation Methods Suitable for Clinical Laboratories. Effect in Cytokine Analysis. Clin Chem Lab Med (CCLM) (2019) 57(10):1539–45. doi: 10.1515/cclm-2018-1297 30990781

[159] Millioni R Tolin S Puricelli L Sbrignadello S Fadini GP Tessari P . High Abundance Proteins Depletion vs Low Abundance Proteins Enrichment: Comparison of Methods to Reduce the Plasma Proteome Complexity. PloS One (2011) 6(5):e19603. doi: 10.1371/journal.pone.0019603 21573190 PMC3087803

[160] Simonsen JB . What are We Looking at? Extracellular Vesicles, Lipoproteins, or Both? Circ Res (2017) 121(8):920–2. doi: 10.1161/CIRCRESAHA.117.311767 28963190

[161] Yuana Y Koning RI Kuil ME Rensen PC Koster AJ Bertina RM . Cryo-Electron Microscopy of Extracellular Vesicles in Fresh Plasma. J Extracell Vesicles (2013) 2. doi: 10.3402/jev.v2i0.21494 PMC3895263 24455109

[162] Yuana Y Levels J Grootemaat A Sturk A Nieuwland R . Co-Isolation of Extracellular Vesicles and High-Density Lipoproteins Using Density Gradient Ultracentrifugation. J Extracell Vesicles (2014) 3. doi: 10.3402/jev.v3.23262 PMC4090368 25018865

[163] Chiam K Mayne GC Wang T Watson DI Irvine TS Bright T . Serum Outperforms Plasma in Small Extracellular Vesicle microRNA Biomarker Studies of Adenocarcinoma of the Esophagus. World J Gastroenterol (2020) 26(20):2570–83. doi: 10.3748/wjg.v26.i20.2570 PMC7265139 32523312

[164] Enciso-Martinez A van der Pol E Hau CM Nieuwland R Van Leeuwen TG Terstappen L . Label-Free Identification and Chemical Characterisation of Single Extracellular Vesicles and Lipoproteins by Synchronous Rayleigh and Raman Scattering. J Extracell Vesicles (2020) 9(1):1730134. doi: 10.1080/20013078.2020.1730134 32158522 PMC7048173

[165] Enciso-Martinez A van der Pol E Lenferink ATM Terstappen L van Leeuwen TG Otto C . Synchronized Rayleigh and Raman Scattering for the Characterization of Single Optically Trapped Extracellular Vesicles. Nanomedicine (2020) 24:102109. doi: 10.1016/j.nano.2019.102109 31669420

[166] Martinez-Castaldi C Silverstein M Bauchner H . Child Versus Adult Research: The Gap in High-Quality Study Design. Pediatrics (2008) 122(1):52–7. doi: 10.1542/peds.2007-2849 18595986

[167] Calin GA Croce CM . MicroRNA Signatures in Human Cancers. Nat Rev Cancer (2006) 6(11):857–66. doi: 10.1038/nrc1997 17060945

[168] Mills J Capece M Cocucci E Tessari A Palmieri D . Cancer-Derived Extracellular Vesicle-Associated MicroRNAs in Intercellular Communication: One Cell’s Trash Is Another Cell’s Treasure. Int J Mol Sci (2019) 20(24):6109. doi: 10.3390/ijms20246109 PMC6940802 31817101

[169] Batchu S Kellish AS Hakim AA . Assessing Alveolar Rhabdomyosarcoma Cell Lines as Tumor Models by Comparison of mRNA Expression Profiles. Gene (2020) 760:145025. doi: 10.1016/j.gene.2020.145025 32758582

