# Supplementary material for: Extracellular Vesicles: A New Source of Biomarkers in Pediatric Solid Tumors? A Systematic Review
Source: Front Oncol. 2022 May 24;12:887210. doi: 10.3389/fonc.2022.887210 (PMC9173703; doi:10.3389/fonc.2022.887210)
Supplement: Supplementary file 1 [file DataSheet_1.pdf]

Supplemental tables

Criteria	Value
Study design	2. Prospective cohort study.<br>1. Retrospective cohort study.<br>0. Other study design.
Patient inclusion	1. Clear and clinically relevant defined inclusion and exclusion criteria.<br>0. Unclear defined inclusion and exclusion criteria.
Patient characteristics	1. Clearly described patient characteristics.<br>0. Unclearly described patient characteristics.
Selection bias	1. No important patient groups excluded.<br>0. Important patient groups excluded or not mentioned/clarified.
Reproducibility	2. Test results are validated in an independent validation cohort.<br>1. Test results are validated in a small* validation cohort.<br>0. Test results are not validated in a validation cohort.
In vitro validation	2. *In vitro* validation of markers with similar EV isolation and characterization methods *in vivo* and *in vitro*.<br>1. *In vitro* validation of markers with different EV isolation and characterization methods *in vivo* and *in vitro*.<br>0. No *in vitro* validation.
End point	1. End point clear at the start of the study.<br>0. End point not clear at the start of the study.
Outcome	2. Clearly described outcome with no missing data.<br>1. Clearly described outcome but with missing data.<br>0. Unclearly described outcome with missing data.
Funding	1. No funding by a commercial entity reported.<br>0. Funding by a commercial entity reported OR no funding reported.

Supplementary Table S1. GRADE scoring with scoring range. *: small cohort: <10 patients; small validation cohort: <50% of the discovery cohort.(1)

Criteria	Score (%)	Description
Nomenclature	5.5 Adherence to MISEV guidelines<br>0 No adherence to MISEV guidelines	Use of operational terms for EV subtypes, unless subcellular origin has been confirmed.
Preanalytical variables	11 Described in detail<br>5.5 Described but not in detail.<br>0 Not described.	Cell culture: characterization of cells, culture and harvesting conditions.<br>Biological fluids: collection conditions and technical details.
Isolation method	11 Reported in detail.<br>5.5 Reported but not in detail.<br>0 Not specified.	Isolation method described, with a level of detail to allow for reproducibility
Source volume & EV abundance	11 Both described quantitatively.<br>5.5 Either described quantitatively.<br>0 Not described quantitatively.	Source: number of secreting cells or volume of biofluid.<br>EV abundance: total particle number and/or protein or lipid content.
EV-enriched proteins	11 ≥3 proteins analyzed.<br>5.5 <3 proteins analyzed.<br>0 Not analyzed.	Positive protein markers of EVs, including 1 transmembrane/GPI-anchored protein and 1 cytosolic/periplasmic protein with lipid or membrane protein-binding ability.
Non-EV-enriched proteins	5.5 Analyzed.<br>0 Not analyzed.	Negative protein marker of EVs.
Antibody & lysis buffer	5.5 Specifics described.<br>0 Specifics not described.	Antibody: reference, provider, clone, and dilution.<br>Lysis buffer: composition and conditions of lysate preparation.
Single vesicle characterization	11 Quantitative and qualitative techniques.<br>5.5 One method for particle analysis.<br>0 No particle analysis reported.	Two different but complementary techniques used with all experimental details for acquisition and analysis reported.
Electron microscopy	5.5 Used/well described.<br>0 Not used.	EM used for evaluation of EV morphology and presence of non-EV structures.
Characterization platform	11 Described in detail.<br>5.5 Described but not in detail.<br>0 Not described.	Platform used to characterize EV cargo reported in detail.
Inclusion of controls	11 Present and described in detail.<br>5.5 Present, not described in detail.<br>0 Not described.	Use of negative/background controls to show that results can attributed to EVs.

Supplementary Table S2. PedEV scores, with detailed scoring range and descriptions.

References to Supplemental Tables:

1. https://www.gradeworkinggroup.org/. GRADE working group.
